# Green approach to synthesis polymer composites based on chitosan with desired linear and non-linear optical characteristics

**DOI:** 10.1038/s41598-024-75953-6

**Published:** 2025-01-24

**Authors:** Dyari M. Mamand, Sarkawt A. Hussen, Shujahadeen B. Aziz

**Affiliations:** 1 Department of Chemistry, College of Science, University of Raparin, Ranya, 46012 Kurdistan Region Iraq; 2 Hameed Majid Advanced Polymeric Materials Research Lab., Department of Physics, College of Science, University of Sulaimani, Qlyasan Street, Kurdistan Regional Government, Sulaymaniyah, 46001 Iraq; 3 Turning Trash to Treasure Laboratory (TTTL), Research and Development Center, University of Sulaimani, Qlyasan Street, Kurdistan Regional Government, Sulaymaniyah, 46001 Iraq

**Keywords:** Chitosan, Green Tea Polyphenol, Metal complex, XRD and ATR-FTIR, UV-vis and optical properties, Chemistry, Materials science, Physics

## Abstract

The current study used sustainable and green approaches to convey polymer composites with desired optical properties. The extracted green tea dye (GTD) enriched with ligands was used to synthesize zinc metal complexes. Green chitosan biopolymer incorporated with green synthesized metal complex using casting technique was used to deliver polymer composites with improved optical properties. The FTIR-ATR was used to identify the functional groups of the GTD, pure CS, and functional groups surrounding the synthesized zinc metal complex. Distinguished ATR bands were observed in green tea dye spectra, such as OH, C = O, and NH functional groups ascribed to various polyphenols. The ATR bands of the zinc metal complex compared to GDT established that GDT is crucial to capturing zinc cations and producing the Zn^2+^-metal complex. The broadness of the bands observed in CS-based composites inserted with the Zn^2+^- metal complex confirms strong interaction among the components of polymer composites. The XRD achievements confirm that CS films with different Zn2+- metal complex concentrations transferred to an amorphous composite. The XRD pattern of composite films establishes that the zinc metal complex scarified the crystalline phases of chitosan. Linear optical properties such as absorption, refractive index (n), and optical dielectric parameters were improved. The absorption edge of the composite’s films shifted to lower photon energies. Various models were used to determine the optical band gap. The band gap drops from $\:5.3\:to\:\approx\:\:1.5\:\text{e}\text{V}$ when chitosan is loaded with a 36% Zn^2+^-metal complex. The Spitzer-Fan method is used to get the dielectric constant, and the Drude Lorentz oscillator model was used to calculate vital optical parameters, including N/m*,* τ*, and *µ*_*opt*_. The W-D single oscillator model was used to determine the *E*_*o*_ and *E*_*d*_ parameters. The values of optical moments (*M*_*−1*_ and *M*_*−3*_) were calculated with the help of the W-D model. The oscillator’s strength ($\:{S}_{0}$) and wavelength ($\:{\lambda\:}_{0}$) were determined via the Sellmeier model using the linear refractive index. The first-order nonlinear ( $\:{\chi\:}^{\left(1\right)}$), second-order non-linear ($\:{\chi\:}^{\left(2\right)}$) and third-order nonlinear optical susceptibility ($\:{\chi\:}^{\left(3\right)}$) were determined for all the films.

## Introduction

Recently, polymer investigations, including structural, optical, mechanical, and electrical, have been of great interest due to the requirements of modern society. The primary reason is that polymers have a wide range of applications. Polymer is a very significant class of materials; without it, one’s life would seem quite tricky by comparison^1^. Polymers’ mechanical, thermal, optical, and electrical characteristics are noticeably lacking compared to metals and ceramics^2^. Multiple techniques have been proposed to improve the optical characteristics and electrical conductivity. One such method is the combination of several types of polymers using salt doping. This allows for the creation of novel materials with various characteristics that would be impossible to achieve with a single metal salt or polymer^3^. Changing the chemical makeup and features of additives at the interface barrier opens up a wide variety of options for the construction of innovative and multifunctional materials. Worldwide energy consumption is projected to double through the second decade of the twenty-first century from its present level of around 16 terawatts^4^. As a result of climate change and carbon emissions, solar power has emerged as a promising renewable energy option that might one day replace fossil fuels^5^. Consequently, the need for advancements in solar technology has grown in the last two decades. Hybrid varieties with inorganic and organic components have supplanted traditional organic solar panels. The innovative method maintains the core advantages of natural energy collecting while using the low-cost manufacturing of organic photovoltaic (OPV) cells^6^. Many semiconductor materials with a direct optical energy gap (DOEG) are used in solar cells and optoelectronic devices^7^. This ideal configuration allows photon-energized electrons to enter the conduction band immediately^8^. Chitosan is a linear polysaccharide biopolymer that is hydrophilic and cationic. It may be readily deduced considering the abundance of natural assets that are readily accessible^9^. One of a kind in terms of its chemical composition, chitosan is a polyamine that carries a positive charge. Biodegradable, non-toxic, Antimicrobial, safe, antioxidant, bio-adhesive, biocompatible, and anti-inflammatory are some of the characteristics shown by this substance^10,11^. Among the many potential applications for these materials are energy storage systems, electrochemical sensors, and biomedical gadgets.

The primary goal of this research is to create films where the amorphous phase predominates; this phase has a greater optical conductivity. Chitosan compound has two reactive functional groups—an amino group at carbon-2 and a hydroxyl group on the backbone and interspersed acetamido groups. Its biodegradability, biocompatibility, and low cytotoxicity have made it worthwhile in biomedical, pharmaceutical, and industrial settings^12,13^. For this reason, chitosan can quickly react with plants like green tea, and metals form coordination bonds. Optoelectronic devices have used polymer complexes as active or passive optical components^14^. Their optical properties determine their potential applications as photochromic materials, optical waveguide materials, photovoltaic cells, field-effect transistors, and high-index-of-refraction films^15^. It is possible to modify the electrical and optical characteristics of polymers by adding dopants. Varied dopants have varied effects on the host material; thus, choosing the right one when incorporating it is essential to get the right property^16^. To implement the above points and improve the optical properties, using chitosan-green tea- Zinc metal and synthesis of complex polymers (CS doped Zn-MC), we were able to pay attention to the specific optical properties of these materials. Several optical devices can be fabricated from it due to the compatibility of the new properties of chitosan. In this paper, we compare the importance of metals on polymer materials in order to improve optical properties, increase light absorption, reduce bandgap energy, and increase non-linear optical properties. The classical Drude-Lorentz model (D.L.M.) and quantum techniques for studying electron transition were covered in the investigation of the principles of light-matter interaction^17^. Compared to more traditional approaches, which use metal nanoparticles or ceramic fillers, the current study uses a low-cost approach to make polymer composites. Since 2019, our research group has produced metal complexes utilizing functional group-enriched tea solutions. The results of the present study establish that green tea dye contains enormous functional groups to capture heavy metals and change them to metal complexes with high optical absorption behavior. Other industries that deal with wastewater contaminated with heavy metal ions will also benefit significantly from this project’s findings^18^. Therefore, this work’s approach to heavy metal ion removal in wastewater will be practical.

Polymer composites with reduced optical band gaps are state-of-the-art in many fields, such as photonics and optoelectronics. The results present in the current study are of high interest due to the high optical performance of the films. The present study opens a new field for research, especially synthesizing metal complexes by tea dyes and its use for polymer composite applications. Combining polar polymers with green synthesized metal complexes can be considered a new field for research and an alternative to traditional methods using ceramic fillers or nanoparticles. The results of the current study are guaranteed to deliver polymer composites with desired optical band gaps using a small amount of metal complexes.

## Materials and methods

### Preparation sample and formation of metal complex

**Scheme 1:**
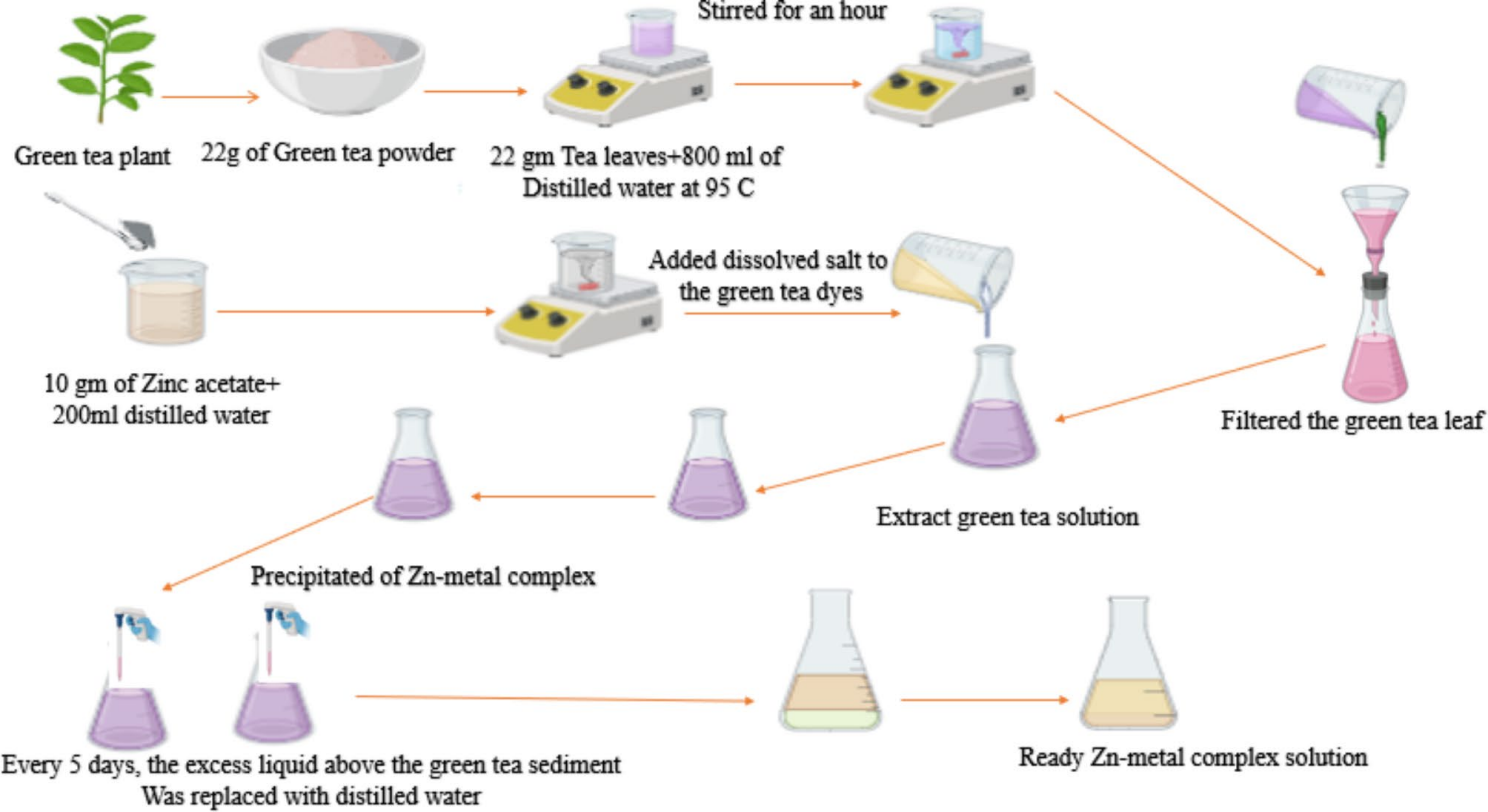
Schematic representation sample preparation method of polymer complex-based chitosan.

Initially, a quantity of green tea leaves weighing 22 g was introduced into a beaker holding 800 milliliters of distilled water at 95 °C. Next, we vigorously swirled the mixture for one hour to ensure thorough mixing. We used paper filtration (Whatman paper 41, cat. no. 1441) with a pore radius of 20 μm to strain the green tea leaf solution. This process effectively eliminated insoluble impurities or undissolved components, resulting in a homogeneous solution. To synthesize Zinc (Zn) metal complexes, 10 g of Zinc acetate were dissolved individually in 200 milliliters of purified water at 70 °C. Subsequently, we introduced dissolved salt into the green tea dyes at 70 °C to form metal complexes. Green tea’s catechins, flavonoids, and polyphenolic compounds can create coordination complexes with metal ions by providing electron pairs (Scheme 1). Green tea has hydroxyl and aromatic groups, making it suitable for ligand use. This is because it may give electron pairs to zinc ions. Consequently, the zinc ion and the green tea polyphenol create a coordination bond. Every polyphenol has many donor sites that bind to a single zinc ion because it donates electron pairs to zinc ions. The process of chelation enhances the stability of the molecule. A precipitate, resulting from the formation of a complex with Zn-metal, was generated and gathered in the bottom of the beaker. The sediment was retained in the beaker, and at regular intervals of five days, any liquid or distilled water that accumulated on top of the green tea sediment was removed using a syringe and replaced with an equal amount of distilled water to remove anions of the dissolved salt and un-coordinated cations of the zinc. The resultant residue was rinsed many times with distilled water.

## Synthesis of polymer composites

**Scheme 2:**
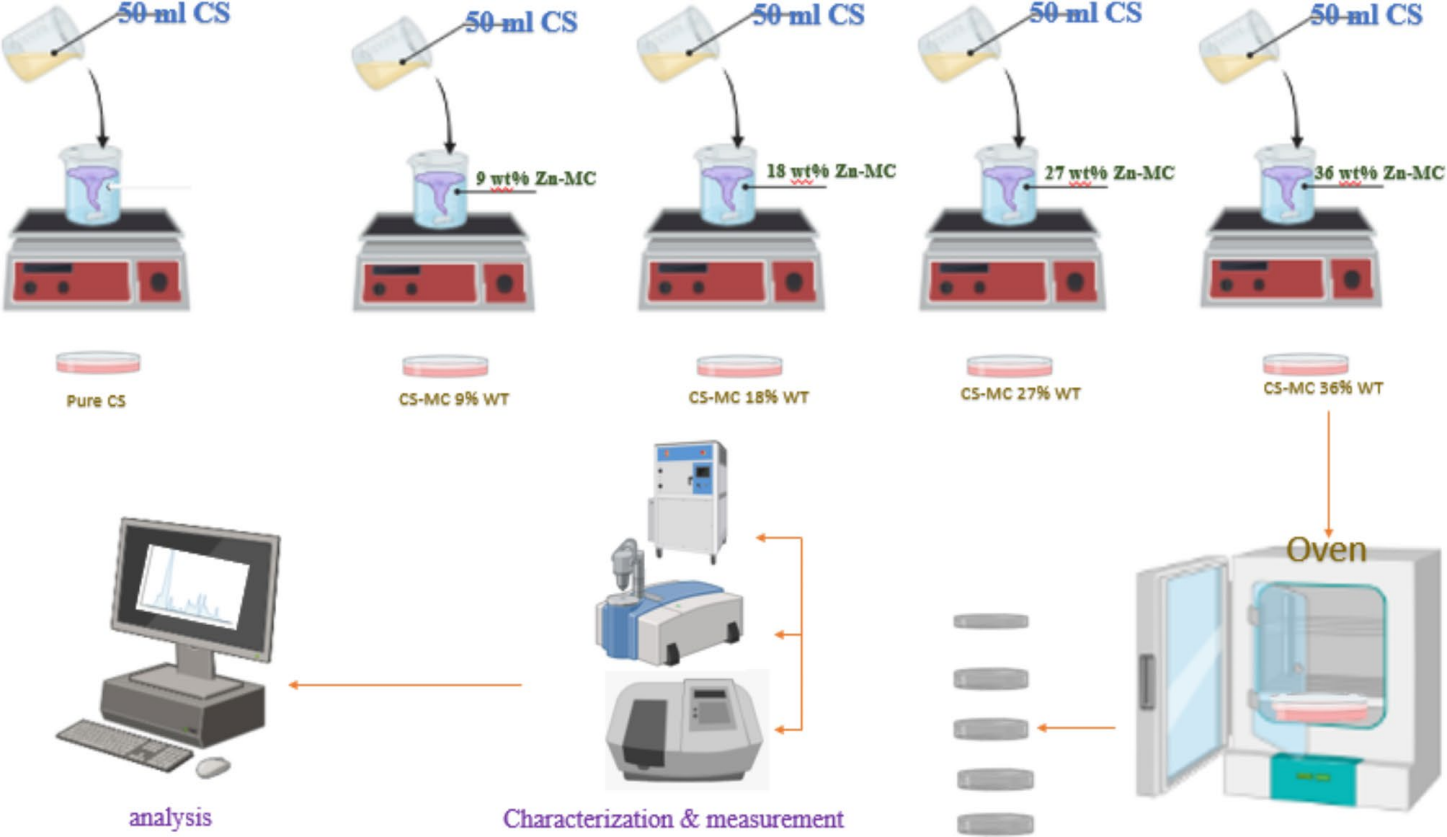
Synthesis of CS: Zn-metal complex procedure to produce freestanding solid polymer composite films.

Polymer composites were created using the solution casting technique, with chitosan biopolymer as the fundamental ingredient. In order to achieve this objective, a 1% solution of acetic acid in distilled water was prepared. Five grams of chitosan were introduced into a 1% acetic acid solution in distilled water while stirring to create polymer solutions. In order to assess the influence of adding a metal complex on the physical and chemical characteristics of the chitosan-complex, four distinct samples were created, each having a different concentration of Zn-metal complex. As shown in the last stage, a specimen of uncontaminated chitosan was produced. After pouring the solution onto a polystyrene petri dish, it was permitted to dry at room temperature to ensure all the solvent evaporated. The film was stored in desiccators with silica gel desiccant to facilitate further drying. The samples are ready for characterization after two weeks. In addition, we assigned the following names to the samples: CSGTDM0, CSGTDM9, CSGTDM18, CSGTDM27, and CSGTDM36. These names correspond to CS solution doped with 0 mL, 9 mL, 18 mL, 27 mL, and 36 mL of Zn-metal complex, respectively. Scheme 2 provides a comprehensive overview of the CS composite fabrication process. The current technology is an environmentally friendly process for manufacturing polymer composites that exhibit excellent film-forming properties.

## Results and discussion

### FTIR analysis

**Fig. 1:**
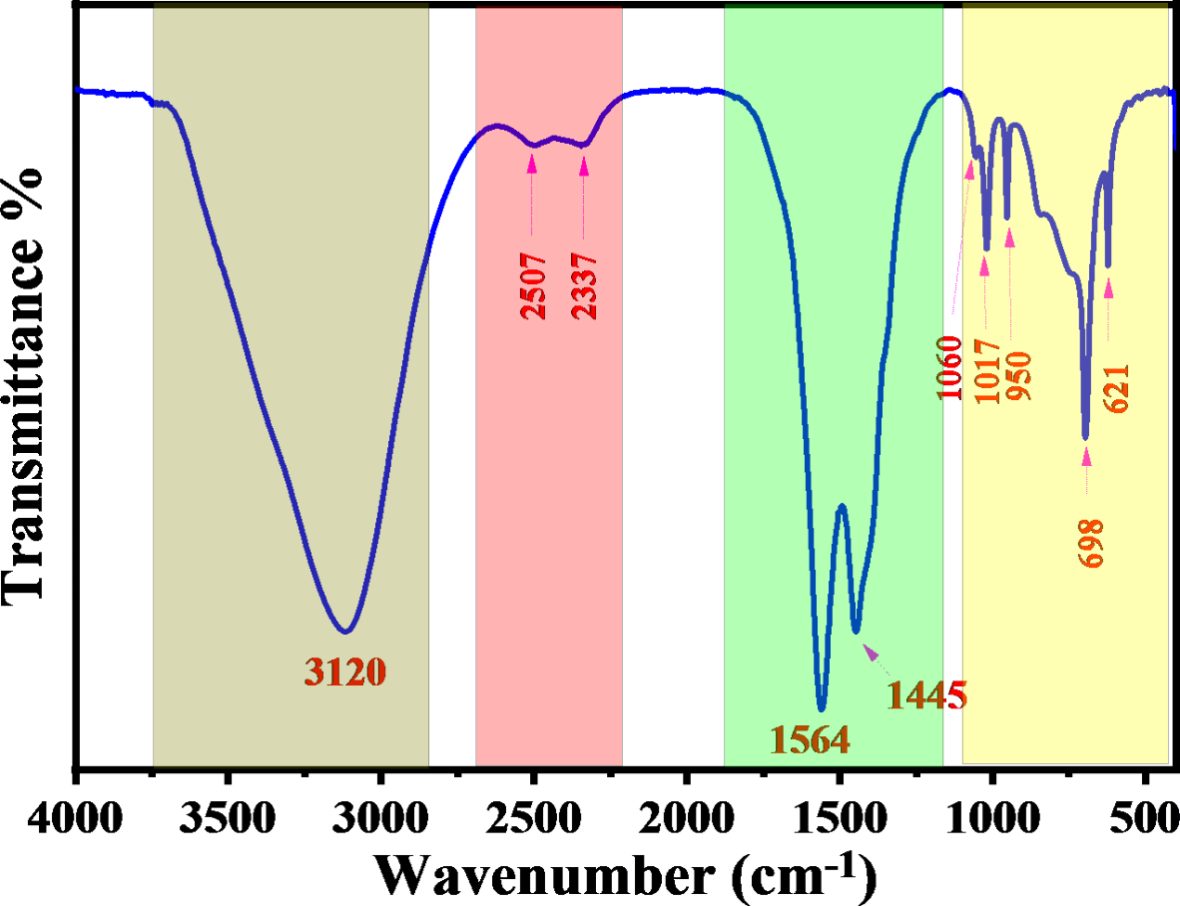
The FTIR spectra for the Zinc acetate salt. The bands due to the anion of the salt ascribed to CH3COO were distinguished at 1564 cm^−1^ and 1445 cm^−1^.

It is possible to qualitatively identify the kinds of bonds present in a sample by comparing the wavenumbers (cm^−1^) of peaks in the absorption spectrum to the vibration frequency of individual molecular parts. The Fourier transform infrared spectrometer typically plots the energy of electromagnetic radiation that passes through the material against a frequency or wave number. The FTIR spectra are captured within the middle infrared spectrum’s 4000 to 395 cm − 1 regions. Figure 1 shows the infrared spectrum of chitosan, which falls into two broad categories, such as the spectra of most organic substances. (The functional group region): 4000 to 1500 cm^−1^. The peaks between 1500 and 500 cm^−1^, also known as the Fingerprint Region, indicate confident types of bonds and may be applied to determining the presence or absence of a particular functional group. Complex molecular deformations give rise to peaks in this area. Such bands could be indicative of molecular symmetry or the result of a mixture of bonds deforming at the same time. The relationship between Zinc ions and green tea ligands and the interaction between metal complexes and chitosan functional groups may be comprehensively analyzed by examining the alterations in the FTIR bands corresponding to certain functional groups. Modifications in the absorption band or the appearance of novel peaks enable the identification of Zinc metal complexes. This information may enhance our comprehension of the binding and coordination chemistry mechanisms. It is fine recognized that the anion of the salt (acetate) is a molecular entity, producing a distinct band in the FTIR spectra. FTIR spectroscopy is a technique that detects and analyzes the vibrations of chemical bonds, allowing for the identification of anions in salts. The identification of the acetate anion group in the compounds is shown by the observation of the last two bands within the 1600 –1300 cm^−1^ range. Figure 1 Examining the FTIR of the salt is essential for understanding the creation of metal complexes.

**Fig. 2:**
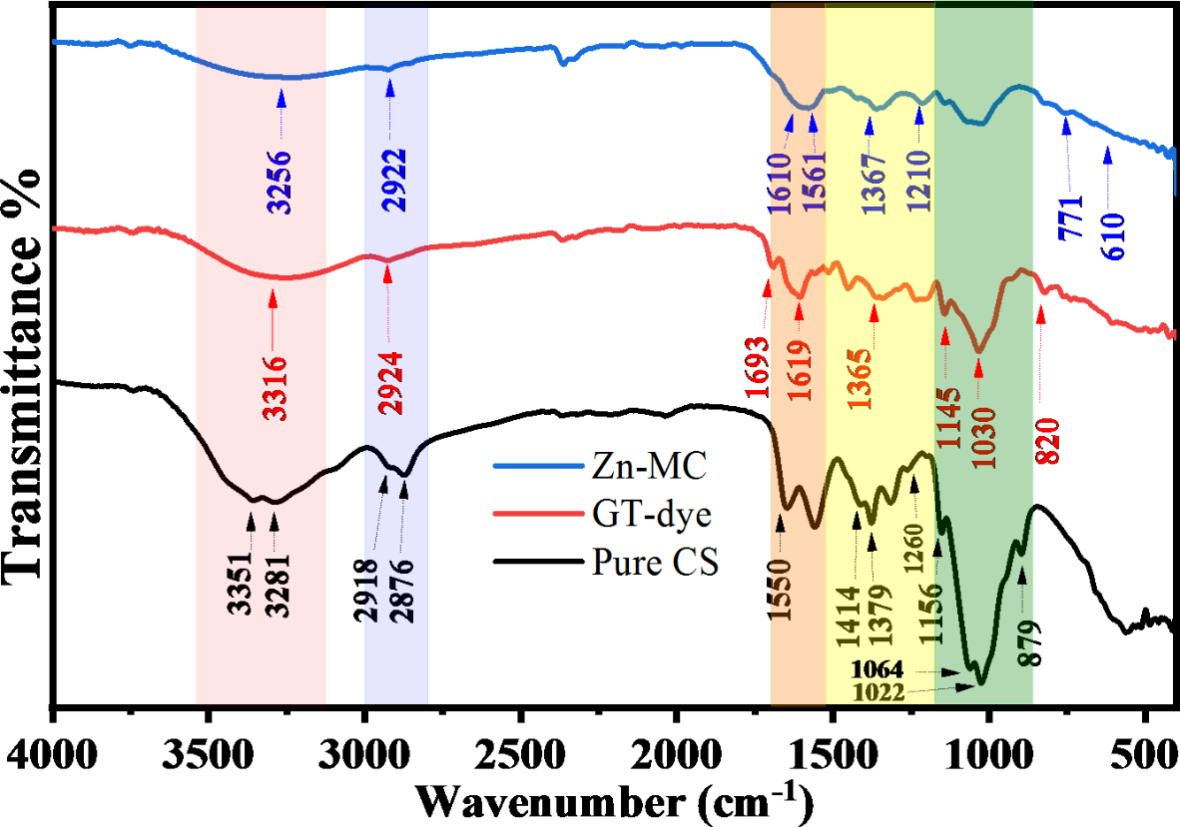
FTIR spectrum of pure CS, GT-dye, and Zn-PPHs.

We can see chitosan’s infrared spectrum, as shown in Fig. 2. The stretching of N-H and O-H bonds, together with the intramolecular hydrogen bonds, are indicated by a prominent band in the 3281 to 3351 cm^−1^ range. The stretching of C-H bonds is responsible for the absorption bands at about 2918 cm^−1^ and 2876 cm^−1^, respectively. Similar bands may be seen in the spectra of various polysaccharides, including^19,20^. The bands at about 1652 cm^−1^ (C = O stretching of amide I) and 1316 cm^−1^ (C-N stretching of amide III) proved residual N-acetyl groups. We did not detect amide II’s N-H bending in the tiny band at 1550 cm^−1 21 ^. Typical N-acetyl groups comprise a third band, which was probable to overlap with others. A band at 1561 cm^−1^ indicates the occurrence of N-H bending in the main amine. The appearance of bands at around 1414 cm^−1^ and 1379 cm^−1^ verified the CH_2_ bending and CH_3_ symmetrical deformations, respectively. The absorption peak seen at a wavenumber of 1156 cm^−1^ may be ascribed to the asymmetric stretching of the C-O-C bridge. The bands seen at 1064 and 1022 cm^−1^ are indicative of C-O stretching. Other researchers have observed the presence of all bands in the spectra of chitosan samples^22^. There is always a chance of contamination involving glycosaminoglycans (GAGs), a different type of polysaccharide in these species. Glycosaminoglycans (GAGs) include sulfate groups covalently attached to the polysaccharide. These sulfate groups may be confirmed in the infrared spectrum by seeing intense bands around 1260–1270 cm^−123^. In the spectrum acquired from chitosan (Fig. 2), the peak at 1260 cm^−1^ is negligible, indicating that it does not originate from sulfate groups. Consequently, it can be concluded that the presence of glycosaminoglycans (GAGs) in the chitosan sample is unlikely. The signal at 1260 cm^−1^ was identified as the bending vibrations of hydroxyl groups found in chitosan. The signal seen at a wavenumber of 897 cm^−1^ correlates to the out-of-plane bending motion of the CH group in the ring structure of monosaccharides^24^.

**Fig. 3:**
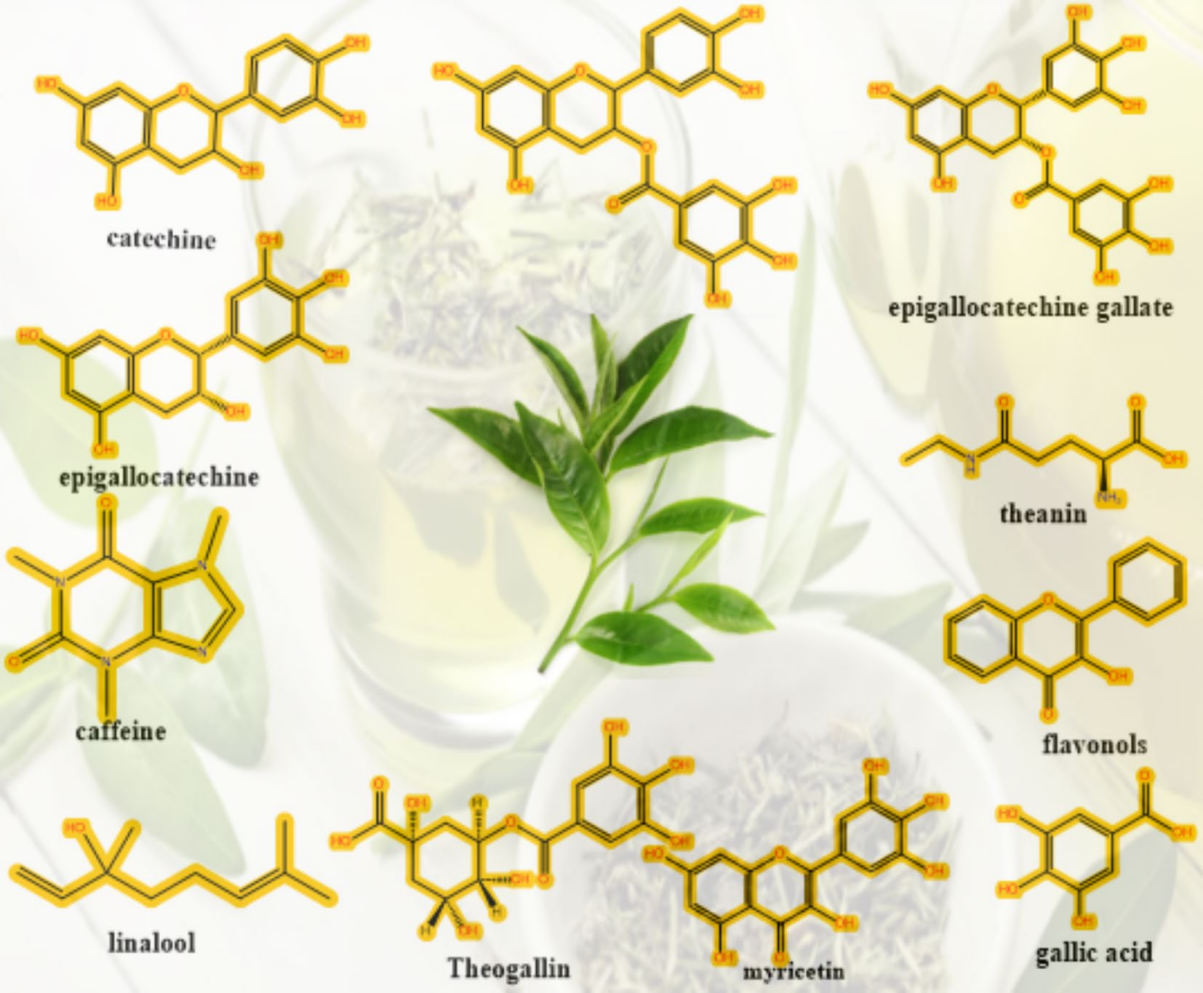
The main components of GT dye are enriched with OH, NH, and C = O groups.

The band seen at 3316 cm^–1^ in the infrared spectra of green tea is attributed to the vibrations that stretch O–H groups, alcohol, and phenols, as well as the N–H stretching in amines. The C–H stretched in alkanes occurs at a wavenumber of 2924 cm^–1^, whereas the O–H stretching in carboxylic acid occurs at 2855 cm^–1^. The intense peak at 1619 cm^–1^ is ascribed to the stretching vibration of the (C = C) carbon-carbon double bond in the aromatic ring and the stretching vibration of the (C = O) carbon-oxygen double bond in polyphenols. The amide-I protein band corresponds to the C–N stretch and is seen at 1365 cm^–1^ wavenumber. The stretching of the C–O–C bonds in polysaccharides results in a peak at 1693 cm^–1^, whereas the stretching of the C–O bonds in amino acids produces a peak at 1030 cm^–1^. The weak band seen at 820 cm^–1^ is attributed to the out-of-plane bending of the C–H bonds. Therefore, the ATR-FTIR spectrum reveals that the green tea sample contains many proteins, amino acids, polyphenols, polysaccharides, and carboxylic acid, as shown in Fig. 3^23^.

**Scheme 3:**
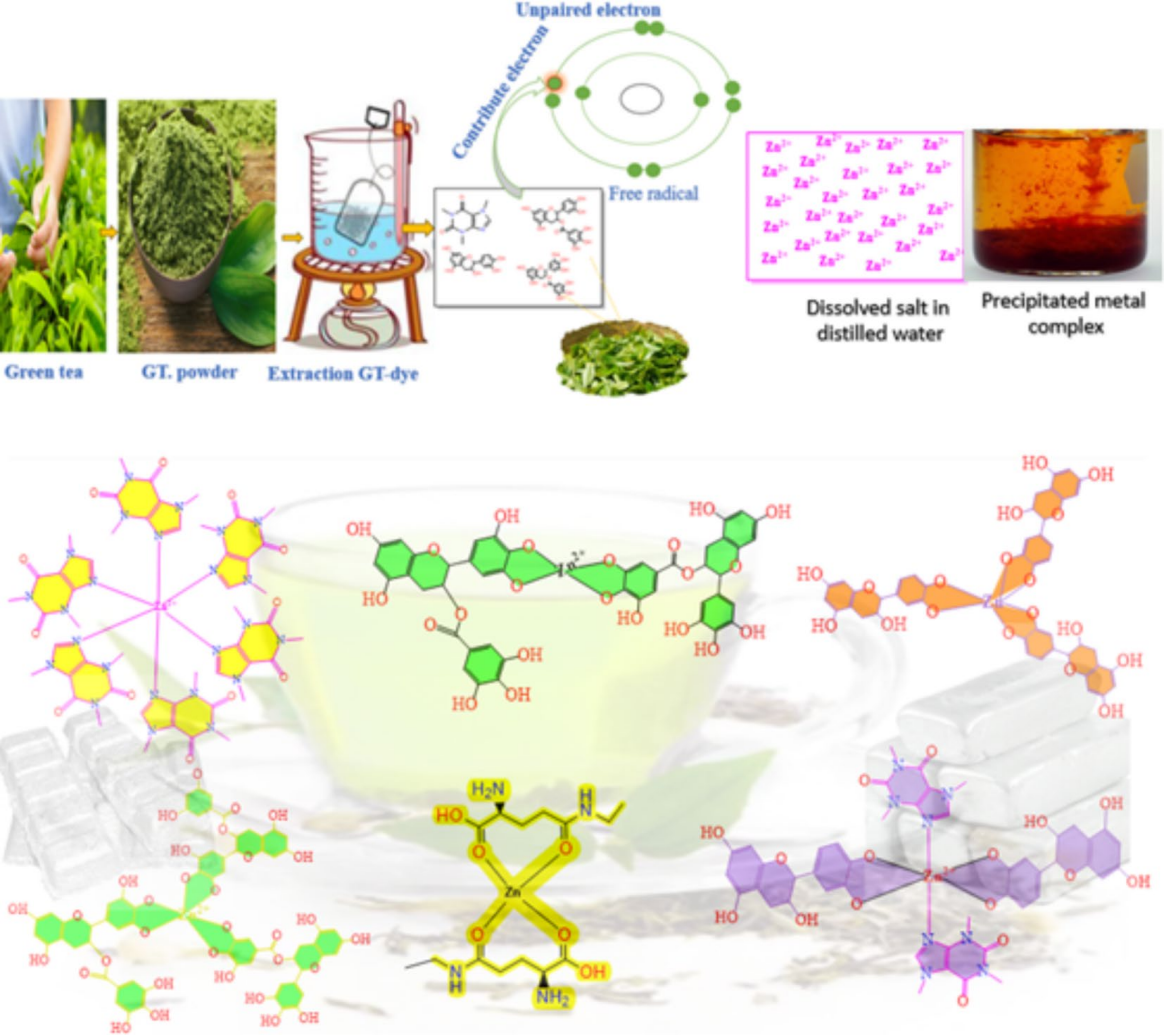
The proposed structure for producing metal complexes involves binding ligands Caffeine and catechin to central metal ions Zn^+2^.

Introducing Zn ions into the GT solution resulted in slight modifications in the FT-IR spectra, the metal-ligand coordination bonds represented by Scheme 3. Specifically, the O–H band changed from 3316 cm^−1^ to 3256 cm^−1^ when the transmittance intensity increased. After adding Zn solution, a new band at 610 cm^−1^ was seen, corresponding to the Zn-O bond^26,27^. The band’s presence at 617 cm^−1^ can be attributed to the vibrations of the metal-ligand complex. Within the framework of zinc complexes, the presence of a band in this specific region frequently signifies the occurrence of Zn-O or Zn-N stretching vibrations. This is in line with the binding of the zinc ion to the O or N (Zn-O or Zn-N) atoms in the green tea dye, specifically in polyphenolic chemicals like catechins. In the aromatic system, the out-of-plane bending vibration band was formed at 771 cm^−1^. Band 1210 cm^−1^ shows the stretching vibration C-O content. In a polyphenol compound such as catechin, it is possible for C-O stretching vibration to be common. When these compounds interact with metals such as zinc, they cause a change in the electron density around oxygen, which causes a shift in the C-O vibrational frequency. The band C-O-C bending or stretching vibrations can be detected in the 1267 cm^−1^ band in the ester and ether groups. The aromatic ring deformation modes may also produce this band; catechins and other compounds with hydroxyl groups as substituents are good examples. The bands at 1561 and 1610 cm^−1^ can be attributed to C = O and C = C stretching in a conjugated aromatic system. Intermolecular hydrogen bonding among polymer chains is disturbed when zinc metal complex is introduced to CS polymers, which means that the amount of intermolecular hydrogen bonding may be reduced. When the zinc metal complex is added to the CS polymer, a strong bond is formed between the hydroxyl OH group of CS and the metal complex’s OH or NH functional groups. The XRD analysis shows that the degree of crystallinity is reduced because this interaction creates a space between these functional groups.

The findings indicate that GT forms compounds with Zinc hydroxyl groups. Alhafez et al. demonstrated that hydroxyl groups in ring B of GT have more negative charges, forming a suitable chelation site^28^. Because polyphenols such as EGCG constitute green tea’s most abundant polyphenol compound, we can hypothesize that PPHs interact with Zn^2+^ and chitosan, as shown in Scheme 3. In summary, the findings indicate that polyphenols form compounds with zinc metal. According to a proposal, the 3-hydroxy group in some polyphenols contains a more acidic proton. This, together with the 4-oxo group, makes it a more favorable location for attaching the metal ion^29^. EGCG has two potential binding sites for the metal ion: the pyrogallol or the galloyl group since it lacks both other groups. According to the findings, the metal ions have a higher affinity for the pyrogallol group of EGCG^28^.

**Fig. 4:**
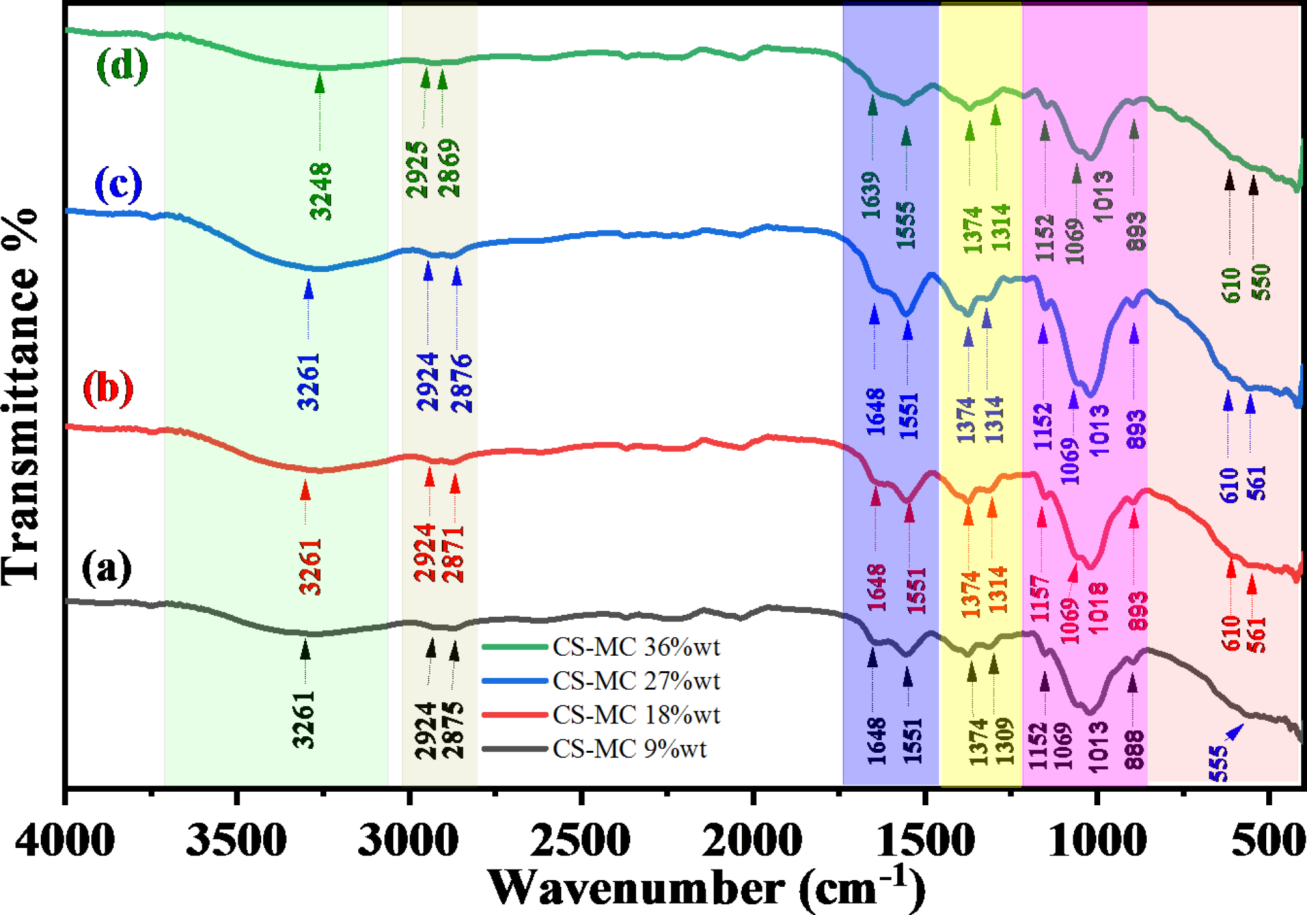
FTIR spectrum CS doped Zn-MC (**a**) 9, (**b**) 18 (**c**) 27, (**d**) 36 wt%.

Reducing the signal at 3361 cm^−1^ indicates a reduction in free -NH_2_ groups, which indicates how they interact with polyphenols and confirms the development of the composite^30^. Furthermore, the peak resulting from the overlapping O–H and N–H stretch moved towards a lower wavenumber of 3276 cm^−1^ in 27% of Zn^2+^-PPHs. This shift indicates a rise in –OH groups due to introducing polyphenols such as catechins on CS, thus giving evidence for developing PPHs-CS, as shown in Fig. 4^31^. These distinctive peaks represent the various functional groups found in chitosan and catechins. Green tea polyphenols were present, according to the FTIR spectra. The existence of a new peak at 1013 cm^-1^ might be attributed to the C-O-C bond of phenols, which would indicate that tea polyphenols were attached to chitosan^32^. In the polymer complex, a peak of 555 cm^-1^ again emerged, which we have indicated is the coordination of zinc with the polyphenols in green tea dye. Zinc can also interact with N (Zn-N) in this range because of an amine group in chitosan. Composite film ATR-FTIR spectra are shown in Fig. 4. As a result of successfully incorporating Zn-GT-dye into the Chitosan matrix, the locations and intensities of distinctive peaks have been adjusted. Composite film spectra show that the interactions between chitosan and polyphenol molecules are linearly enhanced in intensity and band extensiveness as a function of GT. Despite a reduction in intensity, all composite films retain the strong and apparent peak at 1150 cm^-1^, as seen in Fig. 4. The chemical reaction of GT molecules via the pendant groups (-NH_2_ and -OH) of Chitosan is shown by an incremental rise in the intensity of the band at 1539 cm^-1^. Upon the addition of GT, the peak at 1150 cm^-1^ increased and moved to the lower intensity region, suggesting that the presence of GT-PPHs causes modifications in the polymer chains^33^. Some changes in the 3100 to 3500 cm-1 range can be observed, as shown in Fig. 4 for the Zn-MC doped CS. Inadequate crosslinking or bonding may occur at lower concentrations (9%) since the Zn-MC may not bind firmly enough with the chitosan matrix. As a consequence, the broad band of these interactions may be less intense, and the overall influence on the structure may be less noticeable. There could be a point of equilibrium between 18wt% and 27wt% Zn-MC integration into the chitosan matrix, where the ions form stronger interactions like hydrogen bonds or coordination bonds with the functional groups of the chitosan, such as amino and hydroxyl groups. A more pronounced wide band intensity may result from improved structural alterations made possible by this ideal doping. The surface area accessible for interaction with the chitosan is reduced when the Zn-MC starts to agglomerate or form clusters at higher concentrations (36%). The wide band’s intensity might be diminished due to this aggregation, which reduces the effective interaction between Zn-MC and the chitosan matrix. The high concentration of Zn-MC might cause significant structural deformation within the chitosan matrix at 36wt%. The spectral properties and the strength of the wide band might be affected by this distortion, which could decrease the degree of order or crystallinity.

**Scheme 4:**
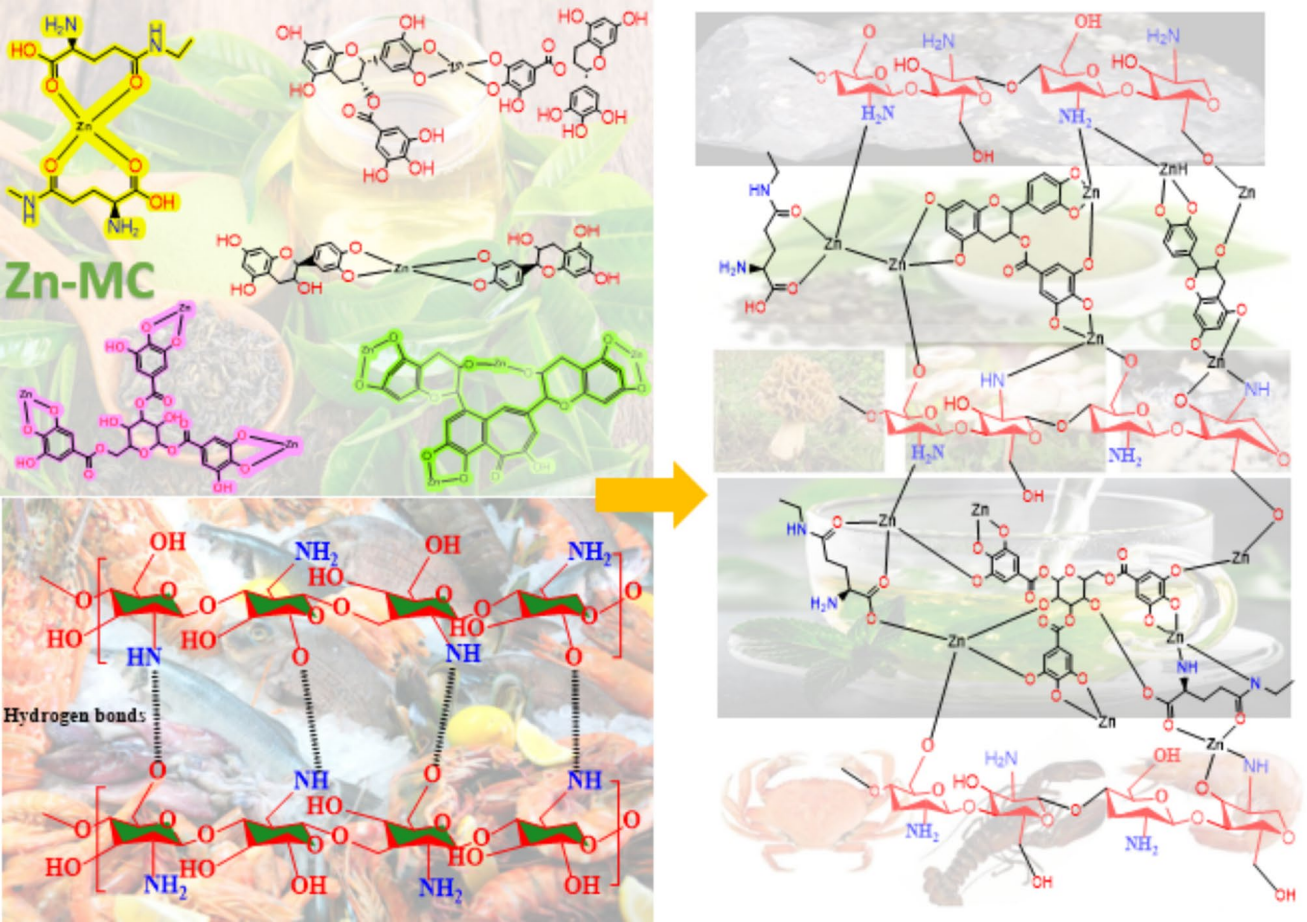
illustrates the process by which the additional Pb-complexes disrupt hydrogen bonding. According to previous research^34–36^ and the current ATR-FTIR spectrum, Scheme 4 is a suggested explanation for how a Zn-metal complex disrupts the hydrogen bonding in the CS host polymer. This complex is chelated or surrounded by different components of polyphenols, specifically primary catechins such as (-)-epicatechin (EC), (-)-epigallocatechin (EGC), (-)-epicatechin gallate (ECG), and (-)-epigallocatechin gallate (EGCG).

#### Scheme 4

Proposed mechanism that illustrates the interaction of Zn-metal complexes with CS backbone. From the scheme it is clear that Zn-complexes interrupt the hydrogen bonding among CS chains. The attachment of the Zn-MC to the functional groups of the CS is reflected in the FTIR spectra in which the intensity of the bands decreased due to the increase of the molecular weight of the resulting product.

## XRD analysis of CS and doped Zn-MC

**Fig. 5:**
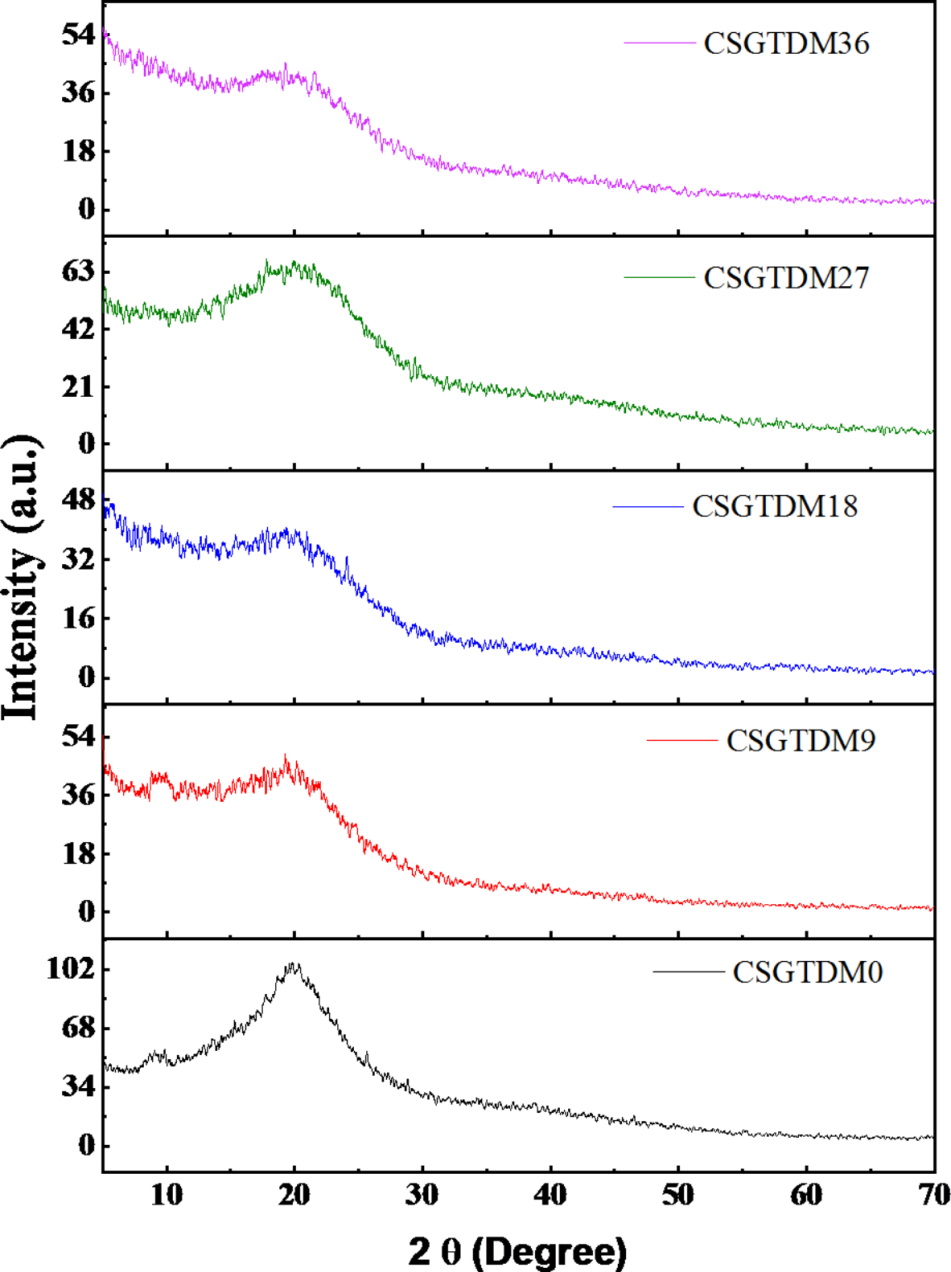
X-ray diffraction spectrum of pure CS and doped metal complex Zn^2+^-MC in different concentrations.

X-ray diffraction (XRD) is a potent analytical tool used to study the crystal structures of materials by detecting the pattern of diffraction generated when X-rays come into contact with a crystalline sample. Chitosan doped with Zn-MC was studied using XRD to learn how the dopants affect the crystal structure of the chitosan matrices. Figure 5 shows that the chitosan control film had a semi-crystalline structure. The control film showed a diffraction peak at 8.75 and 19.8 (2θ), which is consistent with the findings of Mathew et al.^37^ for chitosan films. When Zn-MC was incorporated into chitosan films, the diffraction peak broaded considerably ascribing to amorphous nature of the composites. The intrinsic crystal structure of the chitosan matrix may have been damaged by the interactions among the functional group of CS molecules and the Zn metal complex. Structure disruptions may occur when Zn-MC is present because they change the packing arrangement of chitosan chains. This disorder is caused by disrupting the usual crystalline structure when non-chitosan species, which means Zn-MC, are integrated into the chitosan matrix. The chitosan matrix’s partial amorphization occurred due to a reaction between Zn^2+^-MC and chitosan molecules. Because of their absence of long-range order, which contributes to the noticeable peak widening, amorphous regions are recognized to exhibit broad diffraction peaks in XRD spectra^38^. Interactions between them might lead to structural obligations or chitosan lattice voids. The XRD spectra may show broader peaks if these defects lead X-rays to scatter in various directions^39^. The unequal distribution of the Zn-MC inside the chitosan matrix may cause changes in the local crystal order. An increase in the width of the XRD peaks might be attributed to areas where the chitosan and zinc-metal complexes interact at different levels due to the absence or reduction of multiple crystalline domains with varied sizes and orientations^40^. In XRD characterization, when a peak is narrow and high intensity indicates high crystallinity. This can be seen in pure chitosan, compared to doped Zn-MC. When the doping rate is 9wt%, it is evident that the peak was abroad. With the increase in doping rates, it has become more abroad, as seen in 18wt%, especially when it reaches a high of 36wt%. When the Zn-MC content reaches 27wt%, it may have reached its ideal level of interaction with the chitosan, allowing for enough integration into the chitosan matrix without causing too much disruption. The XRD peak is not as flattened down as it would be at higher doping levels, which means the material keeps more of its original crystalline structure.

### UV-visible spectroscopy

#### Absorption spectrum of CS-GT-MC

**Fig. 6:**
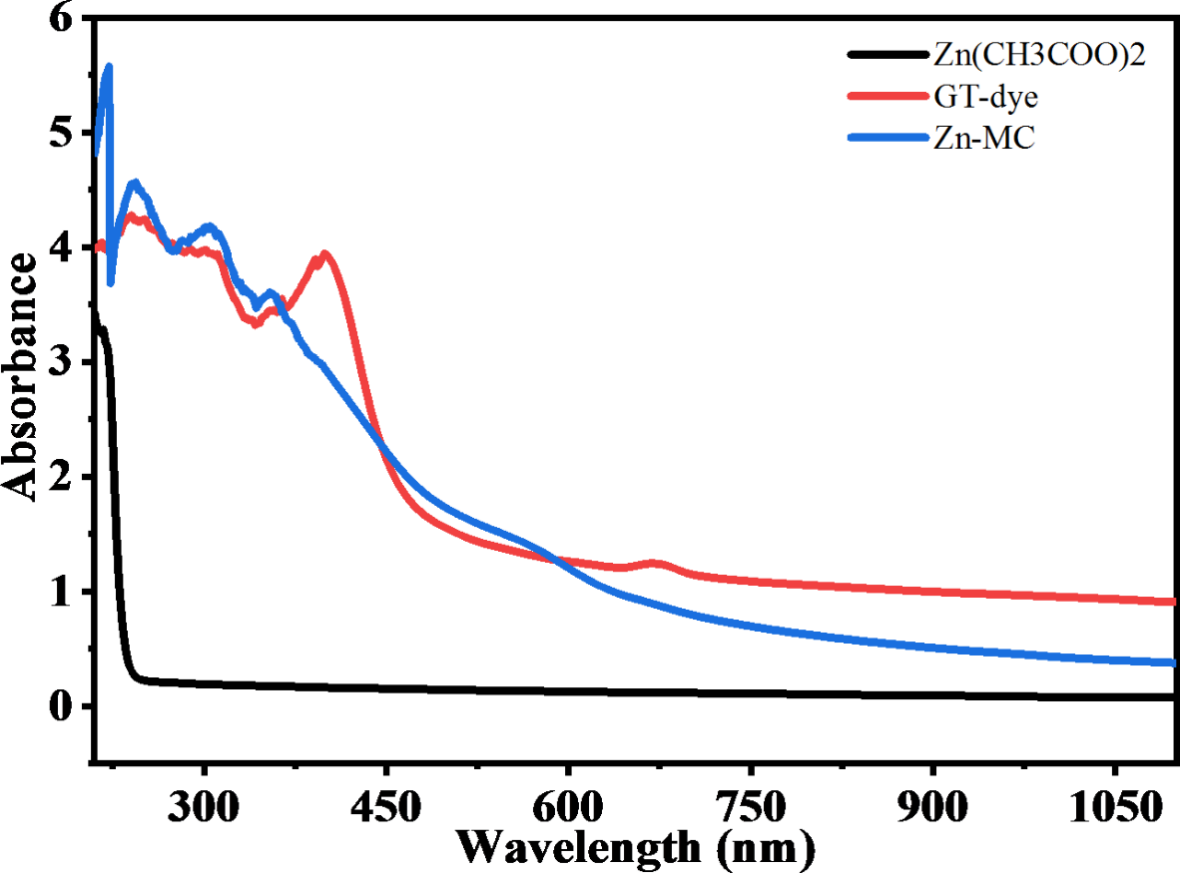
UV-vis absorption of zinc acetate salt, GT-dye and Zn-MC.

Applying UV-Vis spectroscopy, a method of investigation that gives data regarding the absorbance of light at various wavelengths, one may examine changes in absorption bands. It is possible to detect shifts in the electronic and molecular structure of the combination of materials by examining the UV-Vis spectra of the chitosan solution before and after adding Zn-MC. These modifications could be seen as modifications, broadening, and shifts in the intensity of absorption bands. It can be achieved by creating zinc ions (Zn^2^⁺) in a GT-PPHs solution by reacting zinc metal with it, as shown in Fig. 6^41^.

**Fig. 7:**
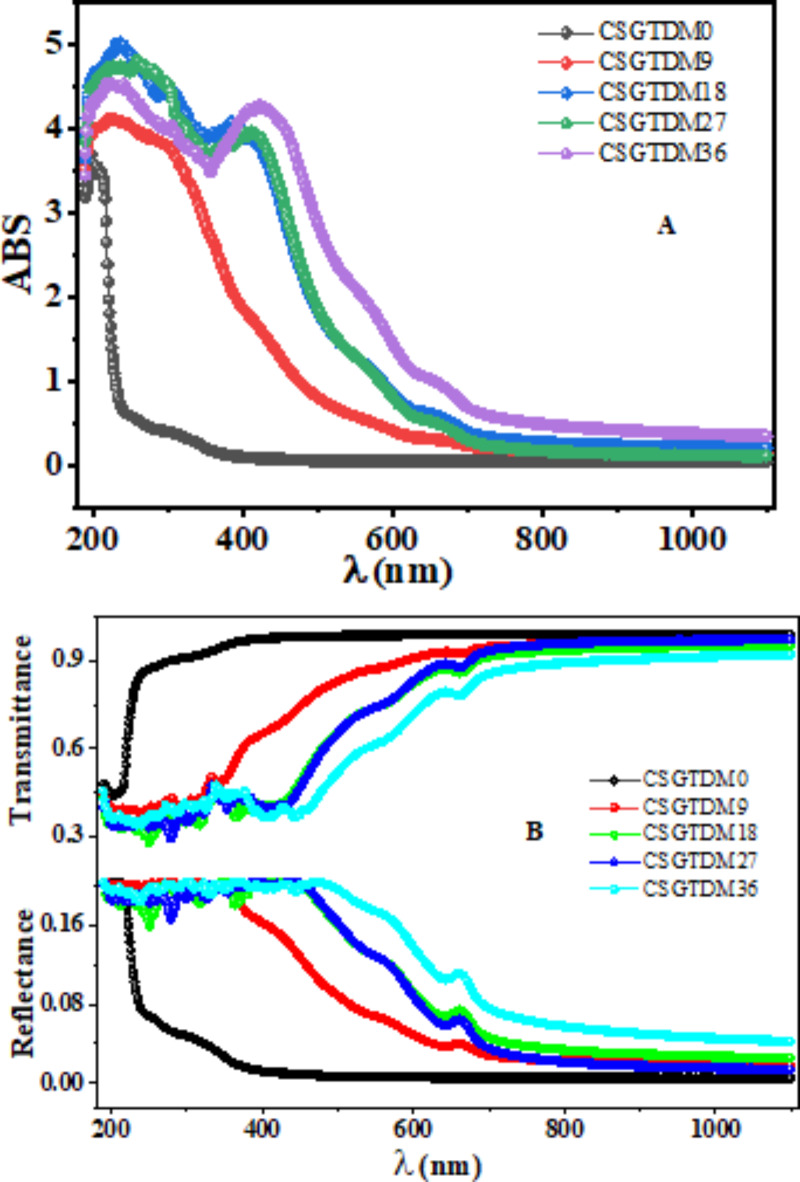
(**A**) UV Absorption spectrum, (**B**) Reflection and Transmittance variations with wavelength of prepared samples CS and doped Zn-MC.

Zinc ions can alter the polymer’s electronic structure by interacting with functional groups found in GT-dye and chitosan, which include amino (-NH_2_) and hydroxyl (-OH) groups^42^. As a result of this interaction, the composite’s UV-Vis spectra show shifted or widened absorption regions, as seen in Fig. 7A. The Zn-MC are recognized to have UV-absorbing capabilities because of the conjugated π-electron groups. Hydrogen bonding and π-π stacking, among other procedures, may allow Zn-MC to interact with chitosan^43^. Absorption regions in the UV-Vis spectra might move or alter due to modifications to the composite’s electronic composition and optical characteristics brought about by these interactions. As shown in Fig. 7A, the concentration of the metal complex caused the absorption band to change completely. Chitosan molecules may undergo charge transfer interactions when Zn-MC present. This has the potential to affect the spectrum features and optical properties of the chitosan polymer by changing the arrangement of electronic states inside it.

The absorption spectra peaks at 324, 363, and 381 nm in the Zn-metal combination are attributed to the transitions from $\:{\uppi\:}\:\text{t}\text{o}\:{{\uppi\:}}^{\text{*}}$ and $\:\text{n}\:\text{t}\text{o}\:{{\uppi\:}}^{\text{*}}$. The major orbital electron pairing is responsible for the lack of the band d-d transition in the Zn-metal complex. Alternatively, the two substances work together to accelerate the bidirectional charge transfer between the ligand and metal. In addition, the potential electronic transition and orbital contributions of ligands and their metal complexes must be carefully considered^42^. Due to the interaction between Zn-MC and chitosan, the electron transition range is shifted to high wavelengths (redshift). The peaks are widely visible in the visible region, as shown in Fig. 7A for different Zn-MC concentrations. Pure chitosan exhibited its maximum wavelength at 203.8 nm. At 18 wt% doping, the peaks were seen at 229 nm and 302 nm wavelengths. At a doping level of 27 wt%, the observed wavelengths were 244 nm, 308 nm, and 376 nm. Peaks were seen at 265 nm and 419 nm, as well as at 231 nm and 449 nm, with a doping concentration of 36 wt%. The absorption in pure chitosan indicates a $\:\pi\:\to\:{\pi\:}^{*}$ transition, this shift from pure chitosan to a longer wavelength indicates the presence of additional electronic transitions caused by Zinc-metal complexes. Interactions between CS and Zn-MC may lead to $\:\pi\:\to\:{\pi\:}^{*}$ transitions in aromatic ligands of the complex, or $\:n\to\:{\pi\:}^{*}\:$transitions in others. A notable redshift at high concentrations for 36wt% doping may be ascribed to robust metal-ligand charge transfer (MLCT) or d-d transitions inside the zinc complex. This phenomenon may result in reduced energy transfer or more conjugation, resulting from enhanced coordination.

It is likely to cause changes to the chitosan polymer’s structure. Among these modifications that could affect its optical characteristics and spectrum behavior include modifications in its conformation, crosslinking density, and molecular weight. As a result of phase transitions occurring in both amorphous and crystalline materials, optical absorption spectroscopy may show how the optical energy bandgap ($\:{E}_{g}$) varies between the conduction and valence bands. Consequently, in the absorption areas of the films containing the complex complexes of polymers^44^. The sample thickness is represented by t, and A is absorbance. T, R, and A can be calculated from the following Eqs. ^45, 46^:

1 $$\:R=1-(T+A)$$

Doping polymer mix complexes may alter their optical characteristics based on their interaction with the host matrix. The impact of Zn-MC doping on optical characteristics is elucidated using the chitosan polymer complex sheets. Figure 7 displays the relationship between optical absorption and wavelength, indicating that absorbance decreases as wavelength increases. This is due to the polymer complex being absorbed at high energies, indicating that a complexation involving the polymer and metal complex has occurred. The energy in this area dictates the bandgap of the samples. Moreover, the absorbance of chitosan-doped Zn-MC-prepared films rises as the MC concentration increases. The intensity of films rises as the concentration of MC increases. This phenomenon occurs because MC has a higher absorption rate than pure Cs. The absorbance curve shows significant shifts at wavelengths between 220 and 310 nm and 310–410 nm, with minor changes in overall intensity around the peak boundaries. The absorption edge undergoes a gradual shift with increasing doping concentration. The absorption of incoming radiation at short wavelengths is due to the combination of Zn-MC with polymer chains, which causes the free electrons to absorb the light. A significant structural alteration in the polymer complex has happened chemically. The appearance of spikes in the uptake of all samples has some possibilities^47^. The cause of the apparent spike is that particle agglomeration or aggregation may cause more light at certain wavelengths to be absorbed^48^.

The interaction of electromagnetic radiation with substances that are absorbing, homogenous, and isotropic is regulated by Beer–Lambert’s equation, and transmission of light spectroscopy is a technology often used in materials research. A comparison of the optical transmittance spectra of MC-doped polymer complex-prepared films with Cs polymer is shown in Fig. 7B. Transparency in the visible range is achieved by the films in their processed state. The graph makes it abundantly evident that the transmission will grow with wavelength when the percentage of Zn-MC in the overall structure of the polymer interact is increased. There is a significant relationship between the size of the surface roughness and the transmission^49^. Considering that the films that have been made are transparent, the rate of transmission is going to increase while the wavelength increases. Materials are entirely transparent in the visible light range and have excellent electrical conductivity^50^. The high reflectance frequently results from the interaction of incident light with free-charge carriers in the substance.

## Tauc plot measurement

An approach widely established and used to analyze the optical absorption edge of materials is known as the Tauc technique^51^. This technique often characterizes working photovoltaic layers, transparent conductors, sensor coatings, and films employed for various functions. First established to determine the absorption characteristics of amorphous film materials, this method was first devised. Consequently, the Tauc approach has garnered considerable popularity for its use on thin crystalline films. Due to their widespread availability, ease of use, and the well-known absorbance equation, UV-Vis spectrometers are often used for the Tauc approach instead of more accurate alternatives like spectroscopic ellipsometry or photothermal spectroscopy^52^. A power-law model is used to fit broad-spectrum absorption spectroscopy observations in the Tauc method. In this approach, the band gap of the substance is determined by where the photon-energy axis intersects. Furthermore, the fit exponent can reveal the direct or indirect nature of the electron transfer. Earlier studies^52,53^, focused on testing how well Tauc’s method for determining band gaps worked. Not included in the study was a determination of how well the Tauc slope factor clarifies the band configuration of an unknown material^53^. The research will statistically analyze the slope values and variations to see if the Tauc approach helps understand the band structure in new systems. Research on the electrical and optical properties of amorphous germanium was carried out by Tauc et al. They presented and backed a method for approximating the band gap from optical absorbance data that is correctly plotted against energy^54,55^. The more extensive work on amorphous semiconductors that Mott and Davis did built upon this idea^56,57^. Their research showed that the photon-to-band-gap energy difference, denoted, determines the optical absorption intensity:

2 $$(ahv)^{\frac{1}{n}}=A^*(hv-E_{g})$$

The following is a list of the variables used in the equation these variables $\:\text{h},\:{\upupsilon\:},\:{\upalpha\:},\:{E}_{g},$ and A* stand for Planck’s constant, photon frequency, absorption coefficient, band gap, and linear area slope, respectively, in the Tauc Plot. The exponent value indicates the characteristics of the electronic transition, such as whether it is permitted or banned and whether it is direct or indirect.

For direct allowed transitions *n* = 1/2.

For direct forbidden transitions *n* = 2/3.

For indirect allowed transitions *n* = 2.

For indirect forbidden transitions *n* = 1/3.

**Fig. 8:**
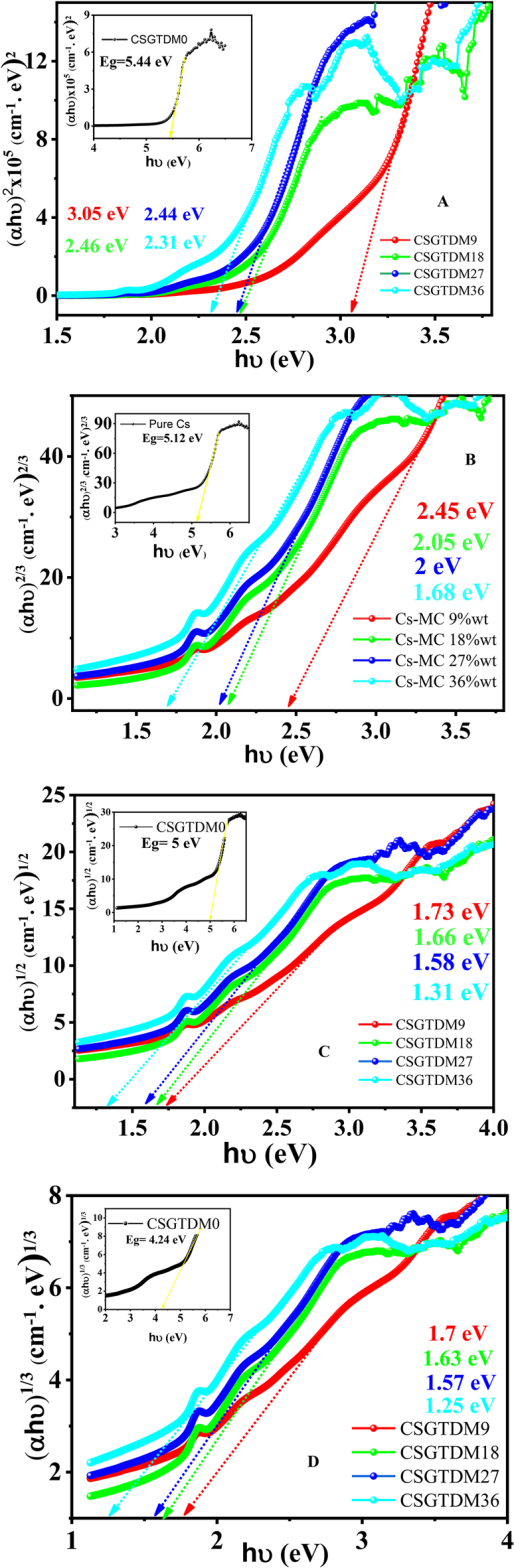
Tauc plot with different value of n of prepared CS doped Zn-MC at various concentrations.

Usually, the permissible changes in energy levels are more influential than the fundamental absorption processes. These changes result in either *n* = 1/2 or *n* = 2, depending on whether the transitions are direct or indirect. The fundamental Tauc analysis process involves obtaining optical absorbance measurements for the specific sample, covering a spectrum of energies from below the band gap transition through above. To plot the (αhv)^1/n^ against ($\:h\nu\:$), one must test several values of n to get the optimal fit. This will help identify the right transition type and determine the band-gap value by locating the intercept on the energy axis. In the indirect scenario, higher energy transitions that can occur by a direct mechanism will consistently exist. In this scenario, extracting indirect and direct connections between data points is feasible by using the suitable exponents in plotting. For example, silicon has an indirect band gap of 1.1 eV, yet it exhibits several intense direct transitions, starting at around 3 eV^58,59^. It is possible to find the intercept (and band-gap) without knowing the slope; hence, the numerical value of A* in Eq. (2) is commonly left out. Displaying the y-axis in relative units alone could be helpful when the film thickness is not specified. However, the bottom absorption processes and band structure are closely connected to the slope, as seen in the next section. The discussion is on the adequacy of normal Tauc plot construction for the precise estimation of important band properties and the unpredictability of experimental data, considering its close connection to band theory, as shown in Fig. 8A, B, C, and D of all samples. Therefore, the next section explains the near-edge residual absorption tail, which can or cannot occur via band-to-band optical transitions. This is the main complicating factor faced when creating Tauc plots.

Zn-MCs involvement may change the $\:{E}_{g}$ of chitosan by introducing new electronic modes into the material’s band structure. Depending on the amount of dopant, the material’s $\:{E}_{g}$ may change. These structural changes may change the material’s bandgap energy and other electrical characteristics. The bandgap has dropped significantly in all the different states, which is considered an achievement. Furthermore, the $\:\pi\:\:\to\:{\pi\:}^{\text{*}}$ electronic transition is responsible for the red shift in the absorption edge ($\:{E}_{g}$)^60^. The electrons are excited as they move from the HOMO to the LUMO in this type of transition. An unsaturated center induces this transition, often in polymers containing double or triple bonds. These results show that the chitosan NH2 and/or OH groups interact strongly with the Zn-MC following doping. The band gap is affected by changes in crystallinity throughout the CS structure, which is similar to this^61^. The results from their XRD and FTIR analyses corroborate this. The exceptionally long straight-chain polymer chitosan comprises glucosamine and N-acetyl-D-glucosamine, randomly linked at β-links. It has a great propensity to form complexes with various ligands and/or metal ions due to the presence of amino, and hydroxyl groups (-NH_2_) and (-OH) groups at certain locations. With the increasing doping concentration of the metal complex, the band gap decreased significantly. The bandgap energy values are shown in Table 1 for all types of transitions, and we can notice many changes because the slope has decreased significantly, proving the method is very successful.

**Table 1:** Optical bandgap energy calculation based Tauc method of CS doped Zn^2+^-MC.

MC-samples	*n* = 2	*n* = 2/3	*n* = 1/2	*n* = 1/3
CSGTDM0	5.44	5.14	5	4.24
CSGTDM9	3.05	2.45	1.73	1.7
CSGTDM18	2.46	2.05	1.66	1.63
CSGTDM27	2.44	2	1.58	1.57
CSGTDM36	2.31	1.68	1.31	1.25

### Refractive index (n) and extinction coefficient (k)

**Fig. 9:**
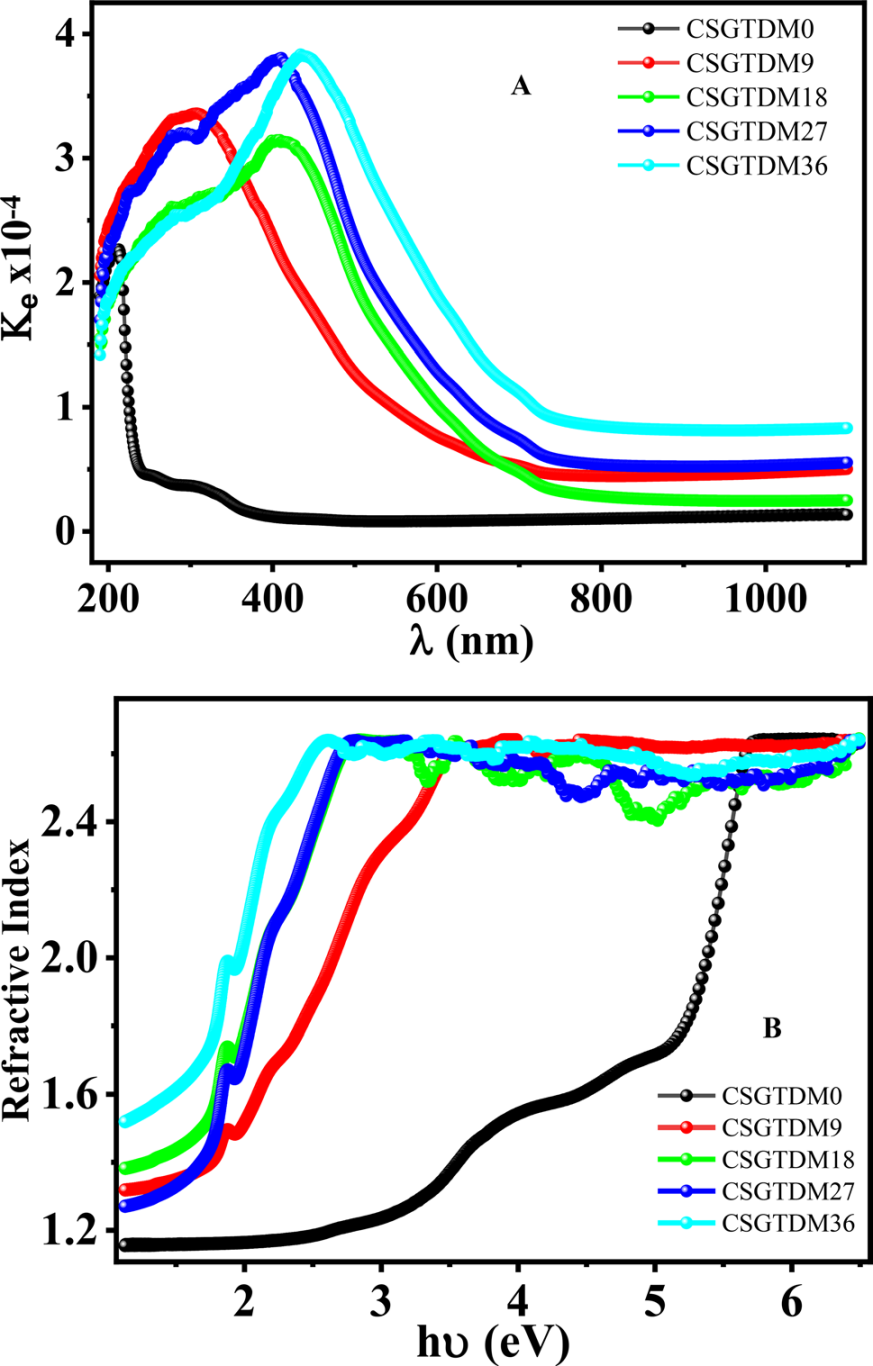
(**A**) Extinction coefficient with wavelength and (**B**) Refractive index and energy variations of pure CS and doped Zn-MC.

The refractive index (n) and extinction coefficient ($\:k$) are essential factors that characterize photonic materials. They are graphed as a function of photon energy in Fig. 9A, B. The refractive index changes minimally in the lower-energy zone and increases in the high photon energy region^62,63^.This pattern can be attributed to variations in the absorption coefficient, causing a spectrum shift in the position of charge polarization owing to inefficiencies in electron energy transfer throughout energy bands. Polymer complex exhibit an exponential increase in the change of their extinction coefficient ($\:k$) depending on the photon energy. This phenomenon occurs when the photon energy is increased. This fluctuation shows that the electromagnetic radiation traveling through the material operates at a higher rate when the photon energy is lower. Because of the growing linear polarization, the Zn-MC concentration in the polymer matrix produced a drop in the whole refractive index estimates. This resulted from reduced atomic refractions induced by the increasing linear polarization, particularly in the ultraviolet region.

The refractive index is a key parameter in electronic components that govern their operation, such as photodetectors, light-emitting diodes (LEDs), and solar cells. Optimal light absorption, minimal reflection losses, and increased device performance are achieved when the refractive indices of the various layers in these gadgets are matched. In complex n calculations $\:\stackrel{\sim}{n}=(n+i{k}_{ex})$, the optical constants are the real and imaginary components. The refractive index, denoted by $\:n$, and the absorption index, or extinction coefficient, denoted by $\:{k}_{ex}$, are both represented by the real and imaginary parts, respectively. The maximum to the lowest transmittance of an electromagnetic beam passing through a polarization optical system equals the extinction coefficient. However, the amount of energy attenuated, degraded, or dissipated by a light beam crossing the thin barrier perpendicularly provides a clearer understanding of the extinction coefficient. An association between the extinction coefficient and the transmittance and absorption curves is established. Looking at the relationship between the extinction coefficient and wavelength, as well as the curves of the transmittance and absorption coefficients concerning wavelength, we can observe that there is minor extinction and no absorption at low absorption. Conversely, when transmittance is high, there is significant absorption and no extinction. As mentioned before, the refractive index of the material represents the proportion of the speed at which light travels in space compared to the speed through the medium that it occupies. The complex refractive index measures this ratio on the opposite end of the spectrum. Due to its effect on the medium’s polarization, the refractive index n is a crucial parameter. The rising refractive index shows the growing polarization of the medium. We may now use the following equations to get the optical constants.

3 $$\:{k}_{ex}=\left(\frac{\alpha\:\lambda\:}{4\pi\:}\right),n=\left(\frac{\left(1+R\right)}{\left(1-R\right)}\right)+{\left[{\left(\frac{\left(1+R\right)}{\left(1-R\right)}\right)}^{2}-{k}^{2}+1\right]}^{0.5}$$

The examined prepared samples’ optical constants’ spectrum dependence upon $\:{E}_{g}$, as shown in Fig. 9A, B correspondingly. A metal complex and CS metal complex optical properties may be explained by two important physical features, such as its refractive index and energy gap. According to Reddy, Herve and Vandame, Kumar and Singh, Ravindra, Tripathy, and Moss, an empirical equation is given below that links the n, and $\:{E}_{g}$ in this context^64^.

Reddy relation:

4 $$\:{n}^{4}\:\left({E}_{g}-0.365\right)=154.$$

Moss relationship:

5 $$\:{n}^{4}{E}_{g}=95\:\text{e}\text{V}$$.

Kumar and Singh relation:

6 $$\:n=K{E}_{g}^{c}$$

Where *K* and *C* are constants and *K* is 3.3668 and *C* is − 0.32234.

Herve-Vandamme relationship:

7 $$\:{n}^{2}=1+{\left(\frac{A}{{E}_{g}+B}\right)}^{2}.$$

Ravindra relationship:

8 $$\:n=4.084-0.62{E}_{g}.$$

Tripathy

9 $$\:{n}_{T}=1.73\times\:\left[1+1.9017\times\:exp-\left(0.539\:{E}_{g}\right)\right]$$

It is clear from the computed results that the gap energy and the refractive index behave in a completely different way, as shown in Table 2. It agrees with the respective research that the refractive index behaves as a function of the gap energy. From all relationships (Reddy, Moss, Kumar & Singh, Herve & Vandamme, Ravindra, and Tripathy), we observed that a decrease in the bandgap energy led to an increase in the refractive index. When *n* = 1/3, it has the lowest band gap at 36wt% of doped Zn-MC compared to other electron transitions and has the highest refractive index. However, when *n* = 2, the maximum band gap and minimum refractive index were observed. We can give this explanation for this purpose. A material’s electronic polarizability, which indicates how readily an external electric field (like light) may distort the electron cloud around atoms, is directly connected to its refractive index. It takes less energy for electrons in materials with smaller bandgap energy to get from the valence band to the conduction band, making these materials easier to excise. Because it is easier to excite, the electrical polarizability is greater, which causes the refractive index to be higher. Additionally, the refractive index shows the degree to which the substance slows down the speed of light. The bandgap of a material determines how many different states its electrons may inhabit when excited. A smaller bandgap material interacts with light more strongly, which causes it to slow down and have a higher refractive index.

Alternatively, as shown in Fig. 9A, B, the extinction coefficient peaks in the UV, drops sharply in the IR and then hits zero in the near-IR. The pattern mentioned above is connected to the absorption coefficient since the light ray dissipation factor is least at regions with a low absorption coefficient. This is associated with an increase in scattering sites and a decrease in transmittance profiles. This study’s refractive index depends entirely on the Zn-MC doping ratio. At low energies, the refractive index increased significantly with increasing Zn-MC concentration. This is due to several factors, including increased density and atomic number compared to pure chitosan. Although light passes progressively across denser materials, a greater refractive index usually indicates a higher density, suggesting a higher concentration of Zn-MC. Increasing the electron density in the pure chitosan matrix affects the polarizability, as the electron density depends on the Zn-MC concentration. The refractive index also increased from 9 wt% to 36 wt%. Adding Zn-MC to chitosan changes the structure of the polymer matrix, which might result in a denser arrangement. As the medium becomes less compressible and more rigid due to its compact structure, the free volume inside the material may be reduced, leading to a higher refractive index.

**Table 2:** Refractive index measurement in different methods associated with different $\:{E}_{g}$ calculated in Tauc method, for CS doped various concentrations Zn-MC.

Sample	Indirect allowed transitions						Direct forbidden transitions					
	Reddy	Moss	K & S	H & V	Ravindra	Tripathy	Reddy	Moss	K & S	H & V	Ravindra	Tripathy
CSGTDM0	2.40	2.08	2.0	1.90	0.98	1.95	2.38	2.07	1.18	1.88	0.89	1.93
CSGTDM9	3.25	2.72	2.82	2.83	3.01	3.02	2.93	2.49	2.11	2.53	2.56	2.60
CSGTDM18	3.30	2.75	2.85	2.86	3.05	3.07	3.09	2.60	2.26	2.68	2.81	2.81
CSGTDM27	3.35	2.78	2.90	2.90	3.10	3.13	3.11	2.62	2.32	2.70	2.84	2.84
CSGTDM36	3.57	2.91	3.08	3.05	3.27	3.35	3.28	2.74	2.61	2.85	3.04	3.06
	Direct allowed transitions						Indirect forbidden transitions					
CSGTDM0	2.34	2.04	1.95	1.83	0.71	1.90	2.51	2.17	2.11	2.04	1.45	2.06
CSGTDM9	2.75	2.36	2.35	2.33	2.19	2.36	3.27	2.73	2.83	2.84	3.03	3.04
CSGTDM18	2.92	2.49	2.51	2.52	2.55	2.60	3.32	2.76	2.87	2.88	3.07	3.09
CSGTDM27	2.93	2.49	2.52	2.53	2.57	2.61	3.36	2.78	2.91	2.91	3.11	3.14
CSGTDM36	2.98	2.53	2.57	2.58	2.65	2.67	3.63	2.95	3.13	3.09	3.30	3.40

### Dielectric properties of CS doped Zn-MC

One of the most critical parameters in creating stable and effective electrochemical devices is the dielectric constant, defined by polymer complexes’ electrical and electrochemical characteristics. Understanding and controlling the dielectric constant is essential for customizing polymer complexes to particular applications in electrochemical sensing, energy storage, and electrical devices^65,66^. It is possible for a polymer that contains doping metal-complexes to demonstrate improved optical and electrical stability and conductivity^67^. When defining the degree of ionic conductivity attained, mechanical durability, and chemical stability of the combination, the bonding involving the molecules of the polymer host and the MC is of the utmost importance^68–70^. The MC low lattice energy is responsible for polymers’ excellent conductivity^71^. The resultant polymer may be more stable because of the reduced lattice energies of the polymers and the doping metal complex. That is why it is preferable for doping MC to have a low lattice energy and a high dielectric constant when they are to dissolve in polymer chains. Applying an electric field causes electric dipoles inside the polymer matrix to align or shift, leading to polarization in polymer complexes. The material’s dielectric constant, electrical conductivity, and energy storage capabilities are all impacted by this polarization^72,73^.

10 $$\:{\epsilon\:}^{*}\left(w\right)={\epsilon\:}_{r}\left(w\right)+{i\epsilon\:}_{i}\left(w\right)$$

Throughout the whole process of light transitioning across the medium, the actual part $\:{\epsilon\:}_{r}$ is determined by the mobility of the electrons. On the other hand, the virtual component $\:{\:\:\epsilon\:}_{i}$ is a representation of the speed at which light departs the material. Therefore, the correlation that occurs may be utilized to acquire the real and imaginary components of the dielectric constant. This can be accomplished by utilizing the quantities n and k obtained^74^.

11 $$\:{\epsilon\:}_{r}\left(w\right)={n}^{2}-{k}^{2}\:$$

12 $$\:{\:\:\epsilon\:}_{i}\left(w\right)=2nk$$

**Fig. 10:**
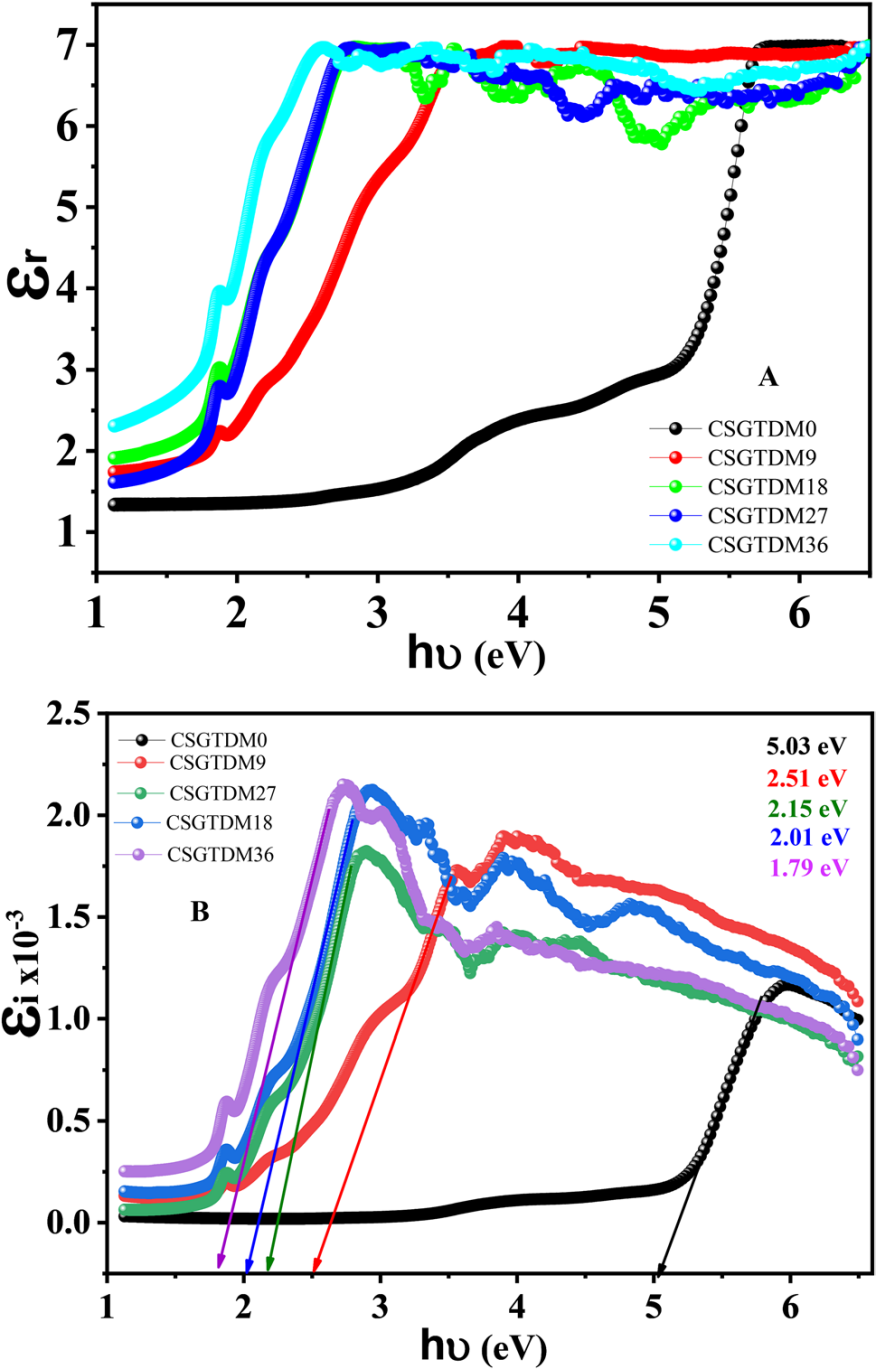
(**A**) real part and (**B**) imaginary part of dielectric constant of pure CS and doped Zn-MC.

At elevated energy levels, such as those associated with smaller wavelengths, the chemical reactions among the components could intensify, resulting in the creation of increasingly enduring complexes and a rise in the value of the dielectric constant, $\:{\:\:\epsilon\:}_{i}\:\text{a}\text{n}\text{d}\:{\:\:\epsilon\:}_{r}$ spectrums are shown in Fig. 10A, B. At elevated energy levels, stronger dipole interactions between molecules may lead to an enhanced polarization ability of the complex and, subsequently, an increase in the actual component of the dielectric constant. Possible explanations for the observed shift in the real part of the dielectric constant from lower to higher energy in the reaction involving the complex Zn-MC include electronic transitions, structural changes, hydration effects, dipole interactions, and complex formation. In this study, the dielectric property was significantly changed by adding metal complexes, especially in the highest, as shown in Fig. 10A, B. Amorphous polymers have randomly arranged polymer chains with no long-range organization. The increased free volume inside the material is usually a result of its disordered structure^75^. The free volume enables quicker ion transport and increases the segmentation mobility of polymer chains. Consequently, ionic conductivity is often more excellent in amorphous polymers than in crystalline ones^76^. A superior dielectric constant is a consequence of this enhanced polarization. The dielectric constant could be increased even more in amorphous areas owing to functional groups with polarity, which can cause dipole-dipole interactions.

### Urbach energy

**Fig. 11:**
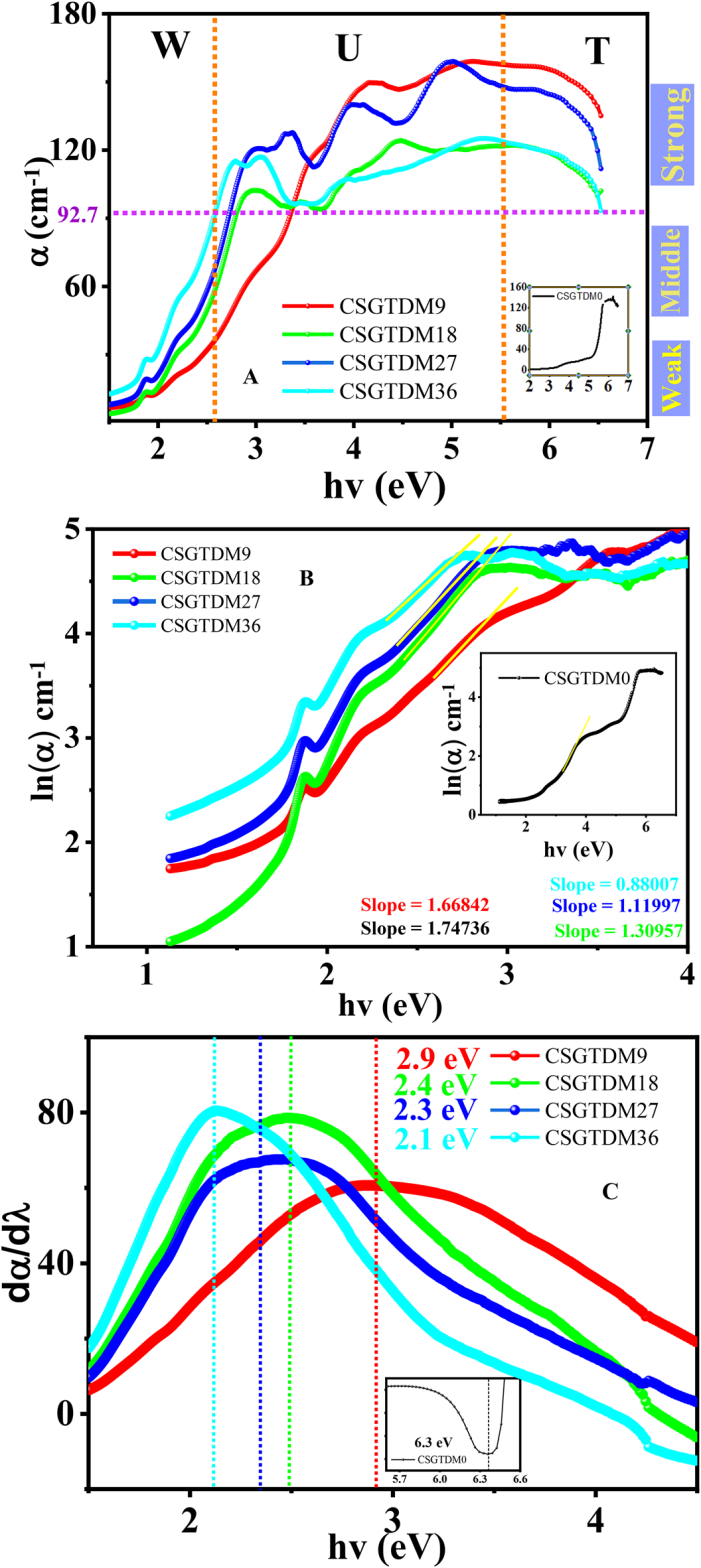

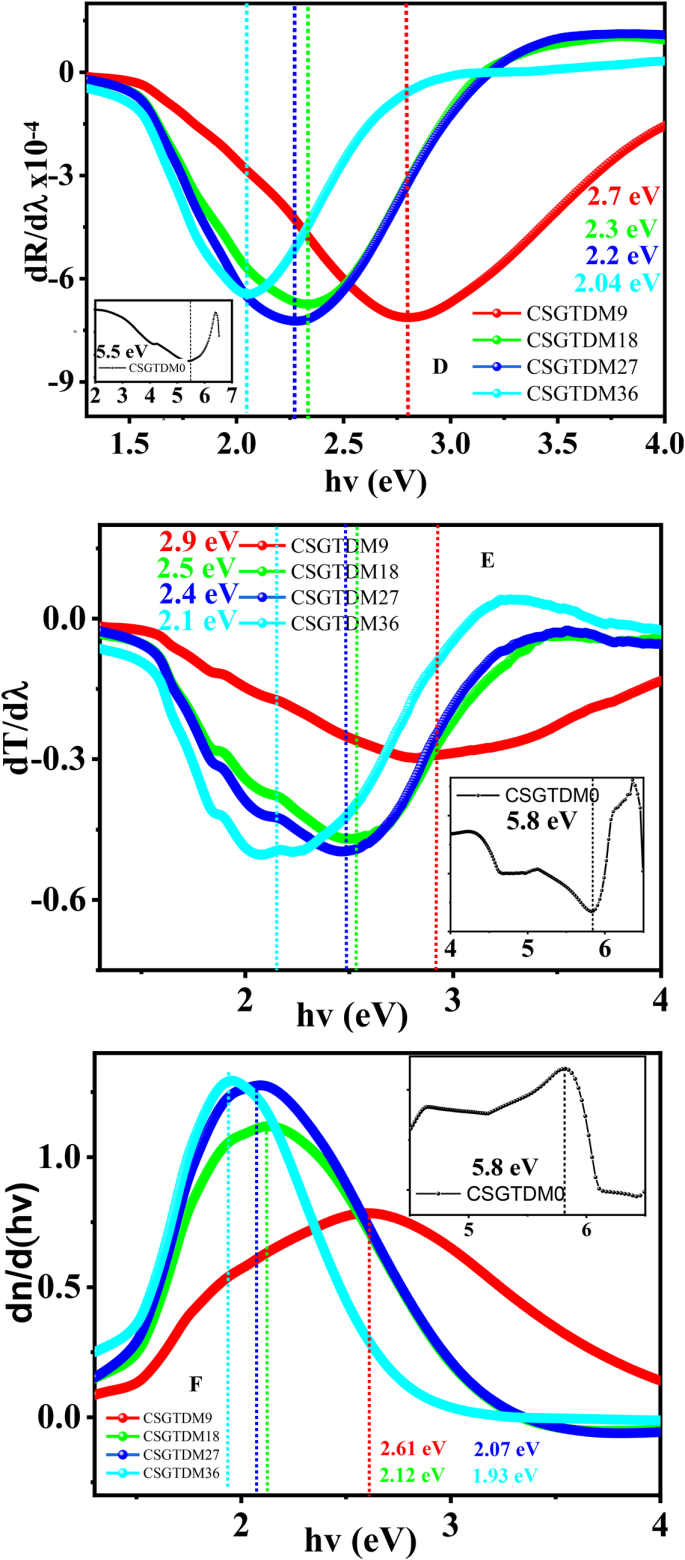
(**A**) variation $\:\alpha\:$ and (**B**) $\:\text{l}\text{n}\left(\alpha\:\right)$ with energy, (**C**) $\:d\alpha\:/d\lambda\:$, (**D**) $\:dR/d\lambda\:$, (**E**) $\:dT/d\lambda\:$, (**F**) $\:dn/d\lambda\:$ with energy for band gap energy calculation of pure CS and doped Zn-MC.

The three levels of optical absorption, strong, medium, and weak, are clearly shown in Fig. 11A, moving from the strongest to the most transparent route. The strong optical absorption zone is separated from the other absorption regions by the horizontal line corresponding to the value (92.7 cm^−1^), which is the optical absorption threshold. Conversely, the optical absorption threshold, which corresponds to the aforementioned horizontal line, is the point at which the absorption curve begins to rise in each prepared sample. Suppose a disorder occurs throughout the process of electrons transitioning through the top edge of the valence band towards the bottom of the band, known as conduction. The electrons come into contact with the density of their corresponding states, denoted as $\:\rho\:\left(hv\right)$, where he represents the energy level of the photon^77^. This leads to the electrons tailing towards the energy gap. The phrase used to describe this portion of $\:\rho\:\left(hv\right)$, that goes towards the energy band gap is the Urbach tail. Therefore, the energy linked to this tail is called the Urbach energy ($\:{E}_{u}$) because it results from the exponential tailing of the absorption coefficient $\:\alpha\:\:\left(hv\right)$. The following equation is used to compute the Urbach energy^78^:

13 $$\:\alpha\:\:\left(hv\right)={\alpha\:}_{0\:}\text{exp}\left(\frac{hv}{{E}_{u}}\right)$$

The photon energy ($\:hv$) and the Urbach energy ($\:{E}_{u}$) are defined in this context, with $\:\alpha\:$ being a constant. As seen in Fig. 11B, the Urbach area is denoted by $\:U$, the weak absorption zone by area $\:w$, and the optical transitions between one extended phase to a different one are denoted by zone $\:\text{T}$. An Urbach tail in area $\:w$ is connected with each specimen. As the MC doping increased, a higher Urbach tailing was seen. According to research, exponential tails are localized state characteristics of weak crystalline surfaces and amorphous disordered materials. Consequently, Urbach tails in area W cannot be produced by electrons transitioning between bands at energies lower around 2.5 eV. This band tailing has been previously attributed to lattice vibration, including antisites, vacancies, and vacancy-interstitial pairs. Because the films’ crystallinity is improved, the Urbach tailing decreases as the MC doped rate decreases. The $\:{E}_{u}$ was calculated first by graphing the logarithm of α against photon energy ($\:hv$) and then fitting a straight line to the linear part of the curve, as seen in Fig. 11B. The films showed smaller crystalline structure, a lower band gap, and a higher Urbach tail as the MC doping increased, this proves that there is a complete agreement between the data of XRD and Urbach Energy. Therefore, all films show a higher $\:{E}_{u}$ than the pure CS because prepared samples contain localized states. Doping with Zn-MC increases the density of localized states close to the conduction and valence bands. In the $\:{E}_{Op}$, these levels may create tails and are prepared to admit electrons. The optical band gap decreases with increasing MC, the surface morphology is homogeneous, and the nanocrystalline material structure has been demonstrated in previous studies on the effects of doping Zn-MC on the optical characteristics, structure, morphology, and microstructure of natural polymer films^79^. The observed narrowing of the optical band gap upon doping may be attributed to the formation of charge transfer complexes within the chitosan host network, which is primarily caused by the incorporation of dopant quantities. The additional absorption peaks in CS carry this view for the doped Zn^2+^-MC samples. We used several methods to calculate the bandgap energy, shown in Fig. 11C, D, E, F.

**Table 3:** The effect of Zn^2+^-MC on Urbach energy, $\:{E}_{g}$ in different methods and $\:{\epsilon\:}_{i}$ of polymer complex-based Chitosan.

films	$\:{E}_{U}$ eV	$\:d\alpha\:/d\lambda\:$	$\:dR/d\lambda\:$	$\:dT/d\lambda\:$	$\:dn/d\left(hv\right)$	$\:{\epsilon\:}_{i}$
CSGTDM0	0.57	6.3	5.5	5.8	5.8	5.03
CSGTDM9	0.6	2.9	2.7	2.9	2.61	2.51
CSGTDM18	0.76	2.4	2.3	2.5	2.12	2.15
CSGTDM27	0.9	2.3	2.2	2.4	2.07	2.01
CSGTDM36	1.13	2.1	2.04	2.1	1.93	1.79

Table 3 used various methods to compare the bandgap energy obtained from the dielectric constant particle imaginer. The results indicate a strong correlation and similarity between the methods. The values of the transitions in the Tauc plot are very close to each other when m = 2/3. This suggests that the type of transition can be classified as direct forbidden transitions.

Because chitosan is a crucial polymer with applications in many different fields, it has been used in many studies and studied in different ways. Table 4 compares the bandgap of chitosan between the different methods to clarify that this method significantly improves the optical properties.

**Table 4:** Effects of different methods and previous studies on the bandgap energy of CS.

MC-complex sample	$\:{E}_{g}/\:eV$	MC-complex sample	$\:{E}_{g}/\:eV$
CS/4% fluorene	5.54^80^	CS/ ZnO NPs	3.26^81^
TiO_2_/CS (75:25)	3.13^82^	CS-MgO nanocomposite	3.4^83^
CS-starch-methyl orange	3.9^84^	CS/SnO_2_ (NPs)	3.54^85^
CS extracted from 2.5 M HCl	3.36^86^	CS/PVP/ 2 wt% hematite	4.5^87^
CS (1 − x): Cuox (4 ≤ x ≤ 12) Nano-composites	3.56^88^	(Cs–Fe) complexes	3.06^89^
CS/3 wt% NiCl2	4.1^90^	CS/3 wt% graphite nanofiller	3.8^91^
TiO_2_/Ag-chitosan composite	4.7^92^	CS-Blend/0.6Ag NPs	4.95^93^

### Dispersion parameters

The Spitzer-Fan method is used to get the dielectric constant. This method uses the equation (N/m*) to determine the dielectric constant; the following is the Eq. ^94^.

14 $$\:{n}^{2}\approx\:{\epsilon\:}_{r}={\epsilon\:}_{\infty\:}-\left(\frac{{e}^{2}\:N}{4\pi\:{c}^{2}{\epsilon\:}_{0}{m}^{*}}\right){\lambda\:}^{2}$$

**Fig. 12:**
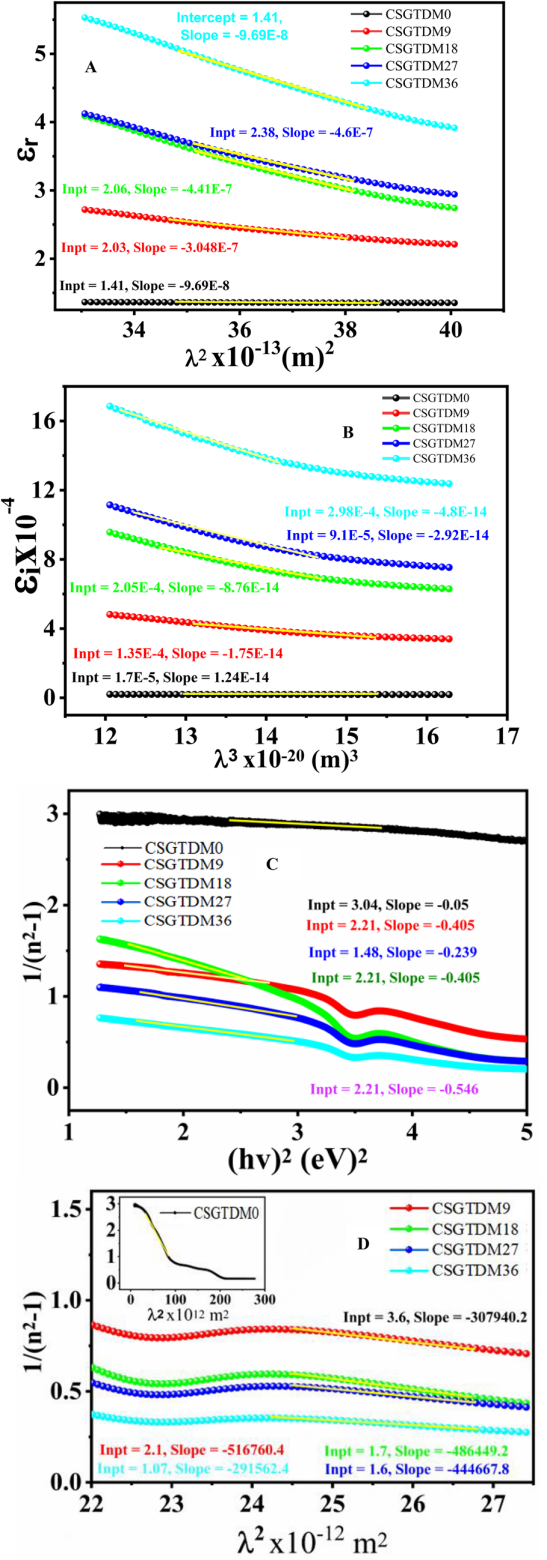
linear region of prepared polymer metal complexes (**A**) $\:{\epsilon\:}_{r}$ against $\:{\left(\lambda\:\right)}^{3}$ (**B**) $\:{\epsilon\:}_{i}$ against $\:{\left(\lambda\:\right)}^{3},\:$(**C**) $\:1/{n}^{2}-1$ against $\:{\left(hv\right)}^{2}$, (**D**) $\:1/{n}^{2}-1$ against $\:{\left(\lambda\:\right)}^{2}$.

At the same time as c represents the speed of light and e represents the charge of an electron, the constant $\:{\epsilon\:}_{0}$ represents the dielectric free space constant. As a result, the $\:{\lambda\:}^{2}$ and $\:{\epsilon\:}_{r}$ values for the pure CS and doped Zn-MC are shown in Fig. 12A. The slope and interception of the straight parts of the diversion are shown in Table 5, calculated from the CS and doped Zn-MC composites, respectively.

**Table 5:** Optical dispersion parameters of Chitosan doped Zn2+-MC.

Films	$\:{\epsilon\:}_{\infty\:}$	$\:\frac{N}{{m}^{*}}{\times\:10}^{52}$	$\:\tau\:\:$ $\:relaxation\:time$	$\:{\mu\:}_{opt}$	$\:{N}_{c}\times\:{10}^{22}$	$\:{\rho\:}_{opt}$	$\:{w}_{p}$
CSGTDM0	1.4	1.17	4.16$\:{\times\:10}^{11}$	6.31$\:{\times\:10}^{23}$	1.25	1.20$\:{\times\:10}^{-27}$	3.41$\:\times\:{10}^{25}$
CSGTDM9	2	3.69	9.26$\:{\times\:10}^{11}$	1.40$\:{\times\:10}^{24}$	3.91	1.72$\:{\times\:10}^{-28}$	1.07$\:\times\:{10}^{26}$
CSGTDM18	2.1	5.36	2.70$\:{\times\:10}^{11}$	4.10$\:{\times\:10}^{23}$	5.67	4.06$\:{\times\:10}^{-28}$	1.55$\:\times\:{10}^{26}$
CSGTDM27	2.38	5.59	1.07$\:{\times\:10}^{12}$	1.62$\:{\times\:10}^{24}$	5.91	9.83$\:{\times\:10}^{-29}$	1.62$\:\times\:{10}^{26}$
CSGTDM36	3.13	9.48	8.66$\:{\times\:10}^{9}$	1.31$\:{\times\:10}^{24}$	10	7.17$\:\times\:{10}^{-27}$	2.75 $\:\times\:{10}^{26}$

**Table 6:** Calculating $\:{E}_{d}$, $\:{E}_{0}$, $\:{n}_{0}$, $\:{M}_{-1}$, $\:{M}_{-3}$ of prepared polymer complex samples-based Chitosan and doped Zn^2+^-MC.

Films	$\:{E}_{d}$	$\:{E}_{0}$	$n_{0}$	$\:{M}_{-1}$	$\:{M}_{-3}$
CSGTDM0	2.43	7.41	1.152	0.32	0.005
CSGTDM9	2.0	3.15	1.278	0.63	0.06
CSGTDM18	1.05	2.33	1.292	0.67	0.10
CSGTDM27	1.67	2.49	1.205	0.452	0.08
CSGTDM36	0.90	2.01	1.204	0.450	0.11

To understand the optical properties of solids, one may use simple equations that connect the complex dielectric function to other quantifiable optical parameters. Prior work established a relationship between the density of localized states of electronics within the forbidden gap of the composite films and the optical dielectric functions ($\:{\epsilon\:}_{r}$ and $\:{\epsilon\:}_{i}$). Through calculating the $\:{\epsilon\:}_{i}$ component with values of N/m*, the Drude free electron theory may readily estimate several more critical features, including plasma frequency ($\:{w}_{p}$), relaxation time ($\:\tau\:$), and optical resistivity ($\:\rho\:$)^95^:

15 $$\:{\epsilon\:}_{i}=J\left(\frac{1}{\tau\:}\right){\lambda\:}^{3},\:J=\frac{{e}^{2}\:\:\:\:\:\:\:N}{8{\pi\:}^{3}{c}^{3}{\epsilon\:}_{0}{m}^{*}}$$

From Eq. (15), we can get the relaxation time ($\:\tau\:)$ variable for every specimen in the area wherein a linear response was achieved for $\:{\epsilon\:}_{i}$ with $\:{\lambda\:}^{3}\:$, refer to Fig. 12B. Equation (14) yielded the N/m* values, and the slope of $\:{\epsilon\:}_{i}$ vs. $\:{\lambda\:}^{3}$ was used to obtain the values of ($\:\tau\:)$. In addition, the following relationships were used to compute the plasma angular frequency ($\:{w}_{p}$), optical mobility ($\:{\mu\:}_{opt}$), and optical resistivity ($\:{\rho\:}_{\text{o}\text{p}\text{t}}$) of the electron^96^:

16 $$\mu_{opt}=\frac{e\tau}{m^*}$$

17 $$\rho_{opt}=\frac{1}{e\mu_{opt}N_c}$$

18 $$w_p=e^2{\frac{N}{\epsilon_0m^*}}$$

Additionally shows the calculated values of $$\:{w}_{p}\:and\:\tau\:$$. The PCs’ relaxation responsiveness to the supplied optical electric field was faster in the occupied PCs compared to the unoccupied ones because the metal complexes decreased the values of $$\:\tau\:$$, $$\:{\rho\:}_{\text{o}\text{p}\text{t}}$$, and $$\:{\mu\:}_{opt}$$ of pure CS. Table 4 shows that when the concentration of Zn-MC increased $$\:{\rho\:}_{\text{o}\text{p}\text{t}}$$ dropped. This is because the number of impurities throughout the band gap decreases the time it takes for electrons to move, resulting from adding additional electron supply sources to the polymer mix. As n increases, the values of $$\:\tau\:$$$$\:{\rho\:}_{\text{o}\text{p}\text{t}}$$, and $$\:{\mu\:}_{opt}$$ fall, leading to a lower light velocity in the surrounding environment. The electron plasma frequency $$\:{w}_{p}$$ rose from $$\:{3.41\times\:10}^{25}$$ to $$\:2.751\times\:{10}^{26}\:Hz$$, a tenfold increase due to the introduction of the Zn-MC. Consistent with previous findings for other polymer complexes, this demonstrates that the metal complexes’ dipole moment increased the material’s polarization in response to the supplied electric field, leading to a strong local electric field. Important for the production procedure for applications involving optics, this led to the discovery of many optical characteristics and the ability to forecast Eg from the optical dielectric loss function97.

To determine whether the films were free of dispersion in refractive index, we compared experimental data using the WDD single oscillator model. Their refractive index and dispersion properties are of greatest importance when studying optical materials. Investigating optical transmission for spectrum dispersion requires careful attention to refractive index dispersion. Research in the normal region may be conducted using the WD single oscillator model^4^. The intensity of the optical transition between the inters bands is measured in this study using a dispersion energy parameter ($\:{E}_{d}$). An important relationship between chemical bonding and the $\:{E}_{d}$ parameter exists because it takes into account the relationship between the coordination amount and distribution of charges in every individual cell. The oscillator energy (average $\:{E}_{g\:}$) is linearly connected to an individual oscillator component ($\:{E}_{0}$). The photon energy and refractive index are linked beneath the inter-band absorption edge, as shown in the essentially empirical expression following^98^.

19 $$\:{n}^{2}-1=\frac{{E}_{d\:}{E}_{0}}{\left[{E}_{0}^{2}-{\left(hv\right)}^{2}\right]}$$

The values of $\:{E}_{0}$ and $\:{E}_{d\:}$ were determined using linear regression lines fitted to the data of $\:1/{n}^{2}-1$ against $\:{\left(hv\right)}^{2}$, using an appropriate slope and intercepts, as well as seen in Fig. 12C. Dispersive energy $\:{E}_{0}$ and single oscillator $\:{E}_{d}$ are the variables in the equation. Table 6 displays the calculated $\:{E}_{d}$ and $\:{E}_{0}$ values. A rise in the energy of dissociation ($\:{E}_{d}$) and a reduction in the energy of binding ($\:{E}_{0}$) were seen when the concentration of the metal complexes increased^99^. When $\:hv\rightarrow:0$, the static refractive index may be found using the Wemple-DiDomenico relation, which simplifies to $\:{n}_{0}^{2}-1=\:{E}_{d}/{E}_{0}$^100^. Zn content is observed to varied as shown in Table 6, which contains the values of $\:{n}_{0}$. So, to explain the $\:{E}_{d}$ chemical composition. development, it could be that the shift in the ionicities and the change in the average coordination number of cations are the main causes of the noticeable variation in dispersion energy values with changing MC content. The previously Wemple-Didomenico single-oscillator factors ($\:{E}_{d}$ and $\:{E}_{0}$) are able to be conveyed using the values of the moments of $\:{\epsilon\:}_{i}$ as follows^101^.

20 $$\:{E}_{0}^{2}=\frac{{M}_{-1}}{{M}_{-3}},\:\:\text{a}\text{n}\text{d}\:\:{E}_{d}^{2}={M}_{-1}^{3}/{M}_{-3}$$

21 $$\:{M}_{-1}=\frac{{E}_{d}}{{E}_{0}},\:and\:{M}_{-3}=\frac{{M}_{-1}}{{E}_{0}^{2}}\:$$

This is because the Wemple-Didomenico single-oscillator variables computation requires the $\:{M}_{-1}$ and $\:{M}_{-3}$ moments from Table 6. Parameter $\:{E}_{0}$, which is an average energy gap, does not affect the magnitude of the imaginary component of the dielectric constant. At the same time, $\:{E}_{d}$ is an inter-band strength variable that relies on the magnitude of the imaginary a component of the dielectric constant. On the other hand, it was previously shown that the (n) at lower frequencies corresponds to the classical dispersion property associated with the single oscillator theory that Sellmeier presented. This feature may be written as follows^97^:

22 $$\:\frac{({n}_{0}^{2}-1)}{{(n}^{2}-1)}=1-{\left(\frac{{\lambda\:}_{o}}{\lambda\:}\right)}^{2}$$

23 $$\:{{(n}^{2}-1)}^{-1}=\frac{1}{({n}_{0}^{2}-1)}-\frac{{{\lambda\:}_{o}}^{2}}{\left({n}_{0}^{2}-1\right){\lambda\:}^{2}}$$

It is essential to substitute the dispersion energy values of $\:{E}_{o}$ and $\:{E}_{d}$ to get the oscillator’s strength ($\:{S}_{0}$) and wavelength ($\:{\lambda\:}_{0}$). Table 7 also contains the results of the correctly determined Sellmeier variables ($\:{\lambda\:}_{0}$ and $\:{S}_{0}$). Furthermore, by rewriting Eq. (25) in the following manner, the relevance of the previous components may have been inferred from a visual representation.

24 $$\:{{(n}^{2}-1)}^{-1}=\frac{1}{({n}_{0}^{2}-1)}-\frac{{{\lambda\:}_{o}}^{2}}{\left({n}_{0}^{2}-1\right)}\frac{{\left(hv\right)}^{2}}{{\left(hc\right)}^{2}}$$

25 $$\:{{(n}^{2}-1)}^{-1}=\frac{{E}_{o}^{2}-{\left(hv\right)}^{2}}{{E}_{d}{E}_{o}}=\frac{{E}_{o}}{{E}_{d}}-\frac{{\left(h\lambda\:\right)}^{2}}{{E}_{d}{E}_{o}}$$

26 $$\:\frac{1}{({n}_{0}^{2}-1)}=\frac{{E}_{o}}{{E}_{d}}\:\text{a}\text{n}\text{d}\:\frac{1}{({n}_{0}^{2}-1)}{{\lambda\:}_{o}}^{2}\frac{{\left(hv\right)}^{2}}{{\left(hc\right)}^{2}}=\frac{{\left(h\lambda\:\right)}^{2}}{{E}_{d}{E}_{o}}$$

27 $$\:\frac{1}{({n}_{0}^{2}-1)}=\frac{{E}_{o}}{{E}_{d}}\:\text{a}\text{n}\text{d}\:\frac{1}{({n}_{0}^{2}-1)}\frac{{{\lambda\:}_{o}}^{2}}{{\left(hc\right)}^{2}}=\frac{1}{{E}_{d}{E}_{o}}$$

28 $$\:{E}_{o}=\frac{\left(hc\right)}{{\lambda\:}_{o}}\:and\:{E}_{d}=\left({n}_{0}^{2}-1\right)\frac{\left(hc\right)}{{\lambda\:}_{o}}\:and\:{S}_{o}=\frac{\left({n}_{0}^{2}-1\right)}{{{\lambda\:}_{o}}^{2}}$$

29 $$\:{{(n}^{2}-1)}^{-1}=\frac{1}{{{{S}_{o}\:\lambda\:}_{o}}^{2}}-\frac{1}{{{S}_{o}\lambda\:}^{2}}$$

Hence, by sketching the link between the two parameters, the refractive index characteristic $\:[{\left({n}^{2}\:-\:1\right)}^{-1}$] and the square of the inverse of the wavelength $\:\left[{\left(\frac{1}{\lambda\:}\right)}^{2}\right]$ one could generate a line that is straight as an illustration of the relation between variables, as shown in Fig. 12D. The line in question meets the vertical axis at a fixed point in the value (1/$\:{S}_{0}{{\lambda\:}^{2}}_{0\:}$), and its slope is proportional to the value (1/So)^102^.

**Table 7:** $\:{S}_{0}$, and $\:{\lambda\:}_{0}$, calculations for investigated samples of polymer complex-based Chitosan.

Films	$\:{S}_{0}\left({m}^{-2}\right)\times\:{10}^{20}$	$\:{\lambda\:}_{0}\left(nm\right)$
CSGTDM0	3.25	176.465
CSGTDM9	1.935	572.965
CSGTDM18	2.06	571.639
CSGTDM27	2.25	299.074
CSGTDM36	3.43	195.71

### Non-linear optical (NLO) properties

Any substance that reacts non-linearly to changes in the strength of the electromagnetic radiation falling on it is said to have non-linear optical (NLO) characteristics. Numerous technical domains, including optic switching, frequency transformation, and communications, place a premium on this quality. Polymer complexes can be used in optical electronics, which has increased fascination with studying their NLO characteristics more than the past few years^103^. New possibilities for practical applications arise when a chitosan-based polymer complex is coupled with Zn-MC; these complexes may show increased NLO characteristics. Polymer complex made from chitosan may have their optical responsiveness modulated by adding Zn-MC, which brings new functions and interconnections. The optical characteristics and chemical structure of chitosan, as well as the structure of the electronic bands of the metal complex made of polymers, can be changed by Zn-MC, inducing quantum confinement effects. In order to fully use these composites in applications related to optics, it is essential to comprehend their non-linear optical characteristics^104^. This research examines polymer complexes based on chitosan and doped with zinc and green tea for their non-linear optical characteristics. Our objective is to shed light on the fundamental processes that control the NLO reaction of these composites. By systematically changing their concentrations, we may learn how Zn-MC contributes to the non-linear optical behavior of the polymer complexes^105^. To go even deeper into the electrical and structural features that impact the NLO characteristics of the mixture of materials, theoretical modeling and computer simulations might be useful^106^. Novel photonic devices with improved functionality and efficiency for uses spanning optical communication towards imaging in the medical field may be built by capitalizing on the unique characteristics of these prepared materials. To find essential parameters of nonlinear optical systems, such as intensity-dependent refractive index, third-order harmonic generation, third-order nonlinear optical susceptibility $\:{\chi\:}^{\left(3\right)}$, and two-photon absorption. The intensity of entering light and the interaction of light waves determine the magnitude of the nonlinear refractive index $\:{n}_{2}$. The phrases that follow illustrate the implementation of experimental formulas for evaluating optical polarizability (P) and nonlinear electron polarizability (PNL)^106^.

30 $$\:P={\chi\:}^{\left(1\right)}E+{P}_{NL}$$

31 $$\:{P}_{NL}={\chi\:}^{\left(2\right)}{E}^{2}+{\chi\:}^{\left(3\right)}{E}^{3}$$

E is the electric field of light, yet first-order nonlinear $\:{\chi\:}^{\left(1\right)}$, second-order non-linear $\:{\chi\:}^{\left(2\right)}$, and third-order non-linear$\:{\chi\:}^{\left(3\right)}$ optical susceptibilities. Also known as n(λ), the refractive index is a.

32 $$\:n\left(\lambda\:\right)={n}_{o}\left(\lambda\:\right)+{n}_{2}\left({E}^{2}\right)$$

Where $\:{n}_{o}\left(\lambda\:\right)\gg\:\:{n}_{2}\left(\lambda\:\right)$, then $\:n\left(\lambda\:\right)={n}_{o}\left(\lambda\:\right)$. $\:{\chi\:}^{\left(1\right)}$ can be written for any medium as

33 $$\:{\chi\:}^{\left(1\right)}=\frac{({n}^{2}-1)}{4\pi\:}$$

Between the relationship of $\:{\chi\:}^{\left(1\right)}$ and $\:{\chi\:}^{\left(3\right)}$ can calculate the $\:{\chi\:}^{\left(3\right)}$ value

34 $$\:{\chi\:}^{\left(3\right)}=\xi\:\:{\left({\chi\:}^{\left(1\right)}\right)}^{4}$$

35 $$\:{\chi\:}^{\left(3\right)}=\frac{\xi\:}{{\left(4\pi\:\right)}^{2}}{\left({n}_{o}^{2}-1\right)}^{4}$$

36 $$\:{\chi\:}^{\left(3\right)}=A{\left[\frac{{E}_{d}}{4\pi\:{E}_{0}}\right]}^{4}$$

For $\:{\chi\:}^{\left(3\right)}$ in (esu), the results obtained from experiments in several previous investigations with various materials have established the constant ξ, represented as 1.7 × 10^−10^. This allows us to use the definition in the reference to get the nonlinear refractive index $\:{n}_{2}$^105^.

37 $$\:{n}_{2}\:=\frac{12\:\pi\:\:{\chi\:}^{\left(3\right)}}{{n}_{o}}$$

**Table 8:** Non-linear optical properties of CS doped Zn^2+^-MC.

film	$\:{\chi\:}^{\left(1\right)}$	$\:{\chi\:}^{\left(3\right)}\times\:{10}^{-16}$	$n_2 \times{10}^{-15}$
CSGTDM0	0.0229	0.467	1.694
CSGTDM9	0.0443	6.547	0.2293
CSGTDM18	0.0468	8.185	0.2856
CSGTDM27	0.0315	1.683	6.015
CSGTDM36	0.0314	1.656	5.922

We can make some suggestions for increased nonlinear optical susceptibility^107^. It is possible that the molecular ordering inside the chitosan matrix is enhanced by its incorporation of Zn-MC. The arranged structure of molecules could become increasingly organized and coherent due to this enhanced molecular ordering. The dipole orientations of chitosan may be increased by substances that include Zn-MC. Excessive dipole moments enhance non-linear optical processes, such as electro-optic phenomena and second harmonic generation, by interacting significantly with an external electric field^108^. In a complex process including chemical reactions, electronic structural modifications, mutually beneficial effects, and molecular ordering, chitosan doped with Zn-MC increases its non-linear optical susceptibilities^109^. Creation and optimization of chitosan-based polymers exhibiting enhanced non-linear optical properties, Table 8 shows that many technological applications depend on a thorough understanding of these chemical factors^110^.

## Conclusion

In conclusion, the solution cast method was employed to synthesize green polymer composites with reduced optical band gaps. The green metal complexes are produced by the ligands of GTD that possess sufficient functional groups to coordinate with the cations of the dissolved salt. Combining CS biopolymers with Zn-metal complexes produced polymer composites with improved optical properties. The crystal structure of the polymer complex was analyzed using XRD. The crystallinity of chitosan decreased substantially as the doping increased. In contrast, the amorphous side increased due to the influence of the metal complex. Chitosan is a semi-crystalline material. Based on the FTIR and peaks, we were able to hypothesize the formation of the structure of this polymer complex. Several optical properties were found in this study, including bandgap energy, which decreased significantly, as well as increased refractive index and optical dielectric properties. We used several other methods to find the bandgap energy and compare them to the optical bandgap energy we saw through the Tauc plot, e.g. $\:dR/d\lambda\:$, $\:dT/d\lambda\:$, $\:dn/d\left(hv\right)$, and $\:d\alpha\:/d\lambda\:$. We computed the $\:{E}_{0}$, $\:{E}_{d}$, $\:{n}_{0}$, and $\:{E}_{0}\approx\:{E}_{g}$. Because the complex material had moved towards amorphous, the Ubach energy went from 0.57 to 1.13 eV, completely compatible with XRD. To further investigate the improvement of the optical properties, we found several parameters, such as relaxation time, optical mobility, and optical resistivity, which we used to compare with other materials. The finding of nonlinear optical parameters also indicates that this method is successful because they increased with the addition of metal to the Zn^2+^-MC complex.

## Data Availability

All data generated or analysed during this study are included in this published article.
